# Smart Mechanoluminescent Phosphors: A Review of Strontium‐Aluminate‐Based Materials, Properties, and Their Advanced Application Technologies

**DOI:** 10.1002/advs.202204925

**Published:** 2022-11-13

**Authors:** Zefeng Huang, Bing Chen, Biyun Ren, Dong Tu, Zhaofeng Wang, Chunfeng Wang, Yuantian Zheng, Xu Li, Dong Wang, Zhanbing Ren, Sicen Qu, Zhuyang Chen, Chen Xu, Yu Fu, Dengfeng Peng

**Affiliations:** ^1^ Key Laboratory of Optoelectronic Devices and Systems of Ministry of Education and Guangdong Province College of Physics and Optoelectronic Engineering Shenzhen University Shenzhen 518060 China; ^2^ Key Laboratory of Artificial Micro/Nano Structure of Ministry of Education School of Physics and Technology Wuhan University Wuhan 430072 China; ^3^ State Key Laboratory of Solid Lubrication Lanzhou Institute of Chemical Physics Chinese Academy of Sciences Lanzhou 730000 P. R. China; ^4^ College of Physical Education Shenzhen University Shenzhen 518060 China; ^5^ Academy for Advanced Interdisciplinary Studies Southern University of Science and Technology Shenzhen 518055 China

**Keywords:** flexible optoelectronics, long afterglow, mechanoluminescence, stress/strain sensing, strontium aluminate

## Abstract

Mechanoluminescence, a smart luminescence phenomenon in which light energy is directly produced by a mechanical force, has recently received significant attention because of its important applications in fields such as visible strain sensing and structural health monitoring. Up to present, hundreds of inorganic and organic mechanoluminescent smart materials have been discovered and studied. Among them, strontium‐aluminate‐based materials are an important class of inorganic mechanoluminescent materials for fundamental research and practical applications attributed to their extremely low force/pressure threshold of mechanoluminescence, efficient photoluminescence, persistent afterglow, and a relatively low synthesis cost. This paper presents a systematic and comprehensive review of strontium‐aluminate‐based luminescent materials’ mechanoluminescence phenomena, mechanisms, material synthesis techniques, and related applications. Besides of summarizing the early and the latest research on this material system, an outlook is provided on its environmental, energy issue and future applications in smart wearable devices, advanced energy‐saving lighting and displays.

## Graphical abstract

Mechanoluminescence materials have recently attracted significant research interest due to their enormous modern applications in energy, sensing, anti‐counterfeiting, wearable display, and sport science. Strontium‐aluminate‐based luminescent materials’ mechanoluminescence is reviewed on the phenomena, mechanisms, material synthesis techniques, and related application.

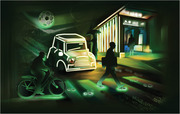

## 1 History of Strontium‐Aluminate‐Based Mechanoluminescence

Mechanoluminescence (ML), a term first proposed by Chandra,^[^ ^1^ ^]^ is the phenomenon in which certain materials emit light under dynamic stress/strain. It may be one of the earliest observed luminescence phenomena other than sunlight by hominids millions of years ago since ML occurs when grinding stones. With the rapid development of photoelectric detection technology and luminescent materials, the field of ML has received extensive attention due to the applications of these materials in various fields such as sensing, imaging, energy, and displays. ML is also referred to by other names, including piezoluminescence, tribophosphorescence, mechanically induced luminescence, stress‐activated luminescence, etc., all of which essentially refer to luminescence under the action of a mechanical force. ML is also classified as triboluminescence and deformation luminescence. Triboluminescence refers to luminescence taking place where two objects are in contact or separated (e.g., luminescence caused by friction between two pieces of mineral quartz). Since the friction between stones emit cold light under a light force, and generate fire under a stronger force, it can be imagined triboluminescence may have inspired the invention of fire in the early stone age, thus marking the beginning of human civilization. Deformation luminescence originates from material deformation, and produces photons below the elastic limit of a material.^[^ ^2^ ^]^ Most mechanoluminescent materials possess the abovementioned two luminescence characteristics, while deformation ML is basically more common in synthetic materials, e.g., SrAl_2_O_4_ (SAO), which is the topic of this review.

SAO‐based materials can emit a wide range of light during afterglow, which is one of the main reasons why it is a well‐known luminescent material since an earlier stage. Among all aluminates, SrAl_2_O_4_:Eu^2+^ (SAOE) is a green phosphor that has been widely studied since the 1960s. Powders of SAOE have a soft green appearance and are widely used in lamps and cathode ray tubes.^[^ ^1^ ^]^ Although the ML of SAOE has been known for more than 40 years^[^ ^1^ ^]^ and was reported earlier in a Japanese patent,^[^ ^3^ ^]^ intensive research only started after the discovery of a bright phosphor with long persistence by codoping of lanthanide ions in 1996.^[^ ^3^ ^]^ In 1996, Matsuzawa et al. obtained intense phosphorescence by cointroducing Dy^3+^ or Nd^3+^ as activators into SAOE and found that Dy^3+^ introduction leads to much better phosphorescence due to its suitable trap depth. Moreover, its brightness and decay time were more than ten times higher than those conventional ZnS:Cu/Co phosphors.^[^ ^4^ ^]^

Multiangle applications and studies of SAOE ML were carried out in 1999 when Xu et al. proposed several material systems. Among them, SAOE was the most widely studied and promising system^[^ ^5^ ^]^ because it is an elastic mechanoluminescent material that has very good crystal resilience and recoverability. In Xu's experiment, an SAOE powder is mixed with an optical epoxy resin and then formed into a disk, strong ML can be observed by the naked eye; the brightness of this resin composite is three orders of magnitude higher than that of quartz.^[^ ^6^ ^]^ In fact, each powder particle of SAOE is a separate luminescent unit and hard to be molded or stressed, so we usually compound it with optical epoxy resin or other matrix to achieve large‐area, high‐sensitivity ML performance.

SAOE has two phases, *α*‐SrAl_2_O_4_:Eu^2+^ and *β*‐SrAl_2_O_4_:Eu^2+^. In 2004, Xu et al. investigated the correlations between ML and the lattice structure of SAOE.^[^ ^7^ ^]^ By controlling the ratios of Sr, Al, and O atoms in the two phases of SAOE, they found that the ratio that obtains the optimal brightness is Sr:Al:O = 1:2:4. The ML performance of *α*‐SrAl_2_O_4_:Eu^2+^ far exceeds that of *β*‐SrAl_2_O_4_:Eu^2+^, which may be due to the different local coordination structures; the former has an asymmetric local field crystal structure, while the latter has an incompletely symmetric crystal structure. In addition, *β*‐SrAl_2_O_4_:Eu^2+^ is characterized by its stronger photoluminescence (PL) compared to *α*‐SrAl_2_O_4_:Eu^2+^. Because of their high symmetry, some other compositions of strontium aluminate, i.e., SrAl_12_O_19_:Eu^2+^, SrAl_4_O_7_:Eu^2+^, Sr_4_Al_14_O_25_Eu^2+^, and Sr_3_Al_2_O_6_:Eu^2+^, nearly exhibit no obvious deformation luminescence due to their high structural symmetry when prepared in a reduction atmosphere and measured in a homemade system. However, the ML of the Sr_3_Al_2_O_6_:Eu^2+[^ ^8^ ^]^ system and the analog Sr_3_Al_2_O_5_Cl_2_:Ln (Ln = Eu^2+^, Tb^3+^, Ce^3+^)^[^ ^9^ ^]^ visible to naked eyes was later discovered by Wang and co‐workers, which indicates that the composite matrix impacts a certain influence on ML, and there may be different ML mechanisms in the highly symmetric structure. Xu and co‐workers compounded Sr_3_Al_2_O_6_:Eu^2+^ ML powders with optical epoxy resin, and observed no deformation luminescence; While in Wang's experiment, Sr_3_Al_2_O_6_:Eu^2+^ ML powders were incorporated into polydimethylsiloxane (PDMS), and the stretching luminescence was observed. Additionally, Wang et al. found that Sr_3_Al_2_O_5_Cl_2_:Ln (Ln = Eu^2+^, Tb^3+^, Ce^3+^) shows no ML when it was compounded with epoxy resin, while weak ML observed for those compounded with silica gel, and stronger ML with PDMS. It can be concluded that the filling organic matrix has a great influence on the luminescence of the inorganic mechanoluminescent powder. Besides, materials prepared in stronger reduction atmospheres have more symmetric crystal structures and exhibit ML properties because of the large number of defects inside the host. The high‐defect state causes the matrix to act as a light‐emitting capacitor, which can store a large number of carriers in excited substates that are capable of supplying energy to the luminous centers to emit light.

## 2 Principles of the Mechanoluminescence of SAOE

**Figure 1:**
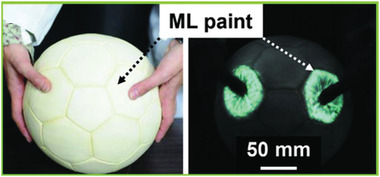
SAOE powder applied to the surface of a soccer ball glows when squeezed. Reproduced with permission.^[^ ^12^ ^]^ Copyright 2017, Fuji Technology Press Ltd.

Under the application of single force, there are two excitation processes for ML deduced from phenomena observed during experiments. One is the release of the carriers in traps. Photons produced by this force‐induced release process will diminish or even disappear after a sufficient number of carriers in the traps are released, which is currently explained by the “piezoelectricity‐induced carrier detrapping model”.^[^ ^10^ ^]^ The other process, photon emission via direct force conversion, is maintained no matter how many times it is repeated within the mechanical fatigue limit. The superposition of these two processes at the microscopic level results in macroscopic luminescence due to the deformation of the material within the elastic limit, which is what we see as “piezoluminescence.” It is important to note that SAOE mainly exhibits the piezoelectricity release type, in which carriers de‐excited by an external force are added to traps over time through high‐energy radiation such as ultraviolet (UV) light or high‐energy particles. Like almost all ML materials, SAOE only responds to dynamic forces/pressure (**Figure** **1**), similar to electromagnetic or triboelectric generation. When a “changing force” is applied, either increasing or decreasing the dynamic pressure will lead to light emission.^[^ ^11^ ^]^

**Figure 2:**
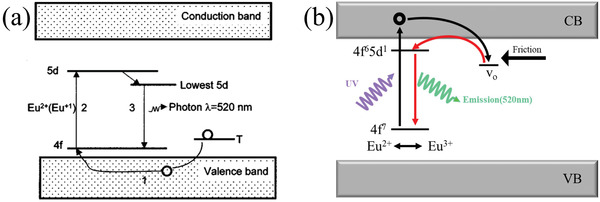
a) Proposed hole carrier model^[^ ^5^ ^]^ and b) modified electron carrier model for SAOE.^[^ ^15^ ^]^ a) Reproduced with permission.^[^ ^5^ ^]^ Copyright 1999, American Institute of Physics. b) Reproduced with permission.^[^ ^15^ ^]^ Copyright 2007, The Electrochemical Society.

Compared with the PL and electroluminescence (EL) processes, the ML process is a more complex dynamic process. It is necessary to consider the mode in which a mechanical force is applied, the conversion process of the force to an electric field/potential energy, the formation process and types of carriers in the material itself, and the energy transfer of doped ions with the luminescence centers. Regarding the carriers involved in the ML of SAOE, there has been debate about whether they are holes or electrons (**Figure** **2**a,b).^[^ ^13^, ^14^ ^]^ In the modified ML process, the f electrons of the Eu^2+^ activators are excited to the conduction band (CB) after irradiation by UV light, releasing an electron and changing to Eu^3+^. The released electron is then trapped at an O defect by crossing the CB. The trap depth usually refers to the energy difference between the energy level of the trap and the top of valence band for hole traps or the energy difference between the bottom of the CB and the energy level of the trap for electron traps. Thermoluminescence (TL) results show that the depth at which trapped electrons are located is 0.2 ± 0.1 eV, so the thermal energy of ≈0.03 eV (350 K) at room temperature is insufficient to release the trapped electrons.^[^ ^5^ ^]^ Electrons captured in SAOE could be released with matching energy to recombine with Eu^3+^ when an external force is applied; then, energy is transferred to Eu^3+^, causing it to change to Eu^2+^ and emit a photon with a wavelength centered at 520 nm. It is noted that mechanically and thermally excited holes are in traps of different depths; ML‐excited holes are usually located in deeper traps, while thermally excited holes are in shallower ones. Thus, the afterglow of the ML material can be observed at room temperature. The atomic radius of Dy^3+^ closely matches the lattice parameter of SAO (close to the atomic radius of Sr), and the introduction of Dy^3+^ ions to SAOE leads to stronger phosphorescence, although the mechanism remains controversial.

**Figure 3:**
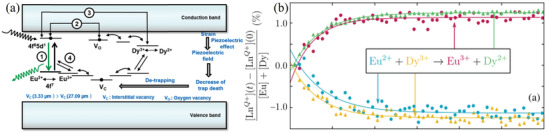
a) Energy band diagram of SrAl_2_O_4_:Eu^2+^, Dy^3+^ (SAOED). Reproduced with permission.^[^ ^16^ ^]^ Copyright 2018, Elsevier Ltd and Techna Group S.r.l. b) Gradual conversion of Eu^2+^ and Dy^3+^ in Sr_4_Al_14_O_25_:Eu^2+^, Dy^3+^ into Eu^3+^ and Dy^2+^ after continuous X‐ray irradiation (60 s in total). It was experimentally confirmed that Dy^3+^ acts as the main electron trap in persistent phosphors, a finding that will help us to further understand the mechanism of Dy in SAOED. Reproduced with permission.^[^ ^18^ ^]^ Copyright 2020, American Physical Society.

In one proposed mechanism (**Figure** **3**a), Dy^3+^ plays a direct role, providing more electron traps.^[^ ^16^ ^]^ In another, Dy^3+^ plays an indirect role (Figure 3b).^[^ ^13^ ^]^ The ionization potential (25 eV) of Eu^2+^ is much smaller than that of Dy^3+^ (41.5 eV), and the ionization potential of Dy^3+^ is smaller than that of Sr^2+^ (43.7 eV). In general, cations have a stronger ability to stabilize O vacancies at a lower ionization potential. Hence, Dy^3+^ will enhance the influence of Eu^2+^ on O vacancies (acting as electron traps), thereby increasing the density and depth of the traps. Regardless of the mechanism, the result is the formation of more shallow traps (as confirmed by TL results^[^ ^4^ ^]^), which contribute to a long‐lasting and pronounced phosphorescence for large numbers of Eu^2+^ and Dy^3+^ ions codoped in aluminates and aluminosilicates^[^ ^13^, ^17^ ^]^ and may also improve ML brightness to some extent. The understanding of ML mechanisms is particularly important for the elucidation of ML characteristics and the rational development and design of materials. It is essential for guiding experiments instead of simple trial and error, reducing test times and synthesis costs, improving efficiency, and continuously improving material properties such as the long afterglow.^[^ ^18^ ^]^

**Figure 4:**
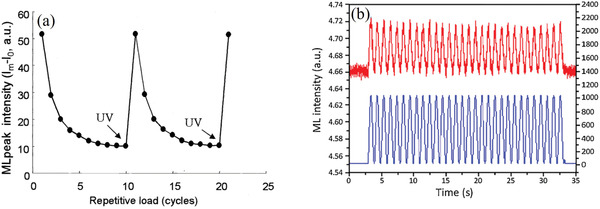
a) Full recovery of the ML intensity by irradiating the sample with UV light at a wavelength of 365 nm. Reproduced with permission.^[^ ^19^ ^]^ Copyright 2001, SPIE. b) Response of an SAOED specimen (compounded with epoxy resin) to a cyclic load at 1 Hz and irradiated with a UV lamp with a power of 1000 mW cm^−2^. Reproduced with permission.^[^ ^21^ ^]^ Copyright 2015, Optical Society of America.

Experiments confirmed that, without energy replenishment, the ML intensity is gradually attenuated during cycles of deformation and the electrons stored in SAOE traps are gradually de‐excited. Further, the ML intensity can be recovered by irradiation from a portable UV lamp (**Figure** **4**a), which replenishes the electrons in traps through continuous exposure.^[^ ^19^, ^20^ ^]^ Moreover, the irradiation time for complete recovery is determined by factors such as the power of the UV light and the thickness of the sample. Kim and co‐workers found that the ML response depends on the loading rate when the light source for excitation is turned off or when a low‐power light source is used. By contrast, the ML response does not depend on the loading rate when a high‐power excitation source is used but only depends on the magnitude of the applied load. Good ML repeatability makes it possible for SAOED to achieve repeatable and accurate detection of stress and strain (Figure 4b).^[^ ^21^ ^]^

## 3 Improvement in Mechanoluminescence for SAOE

### 3.1 Brightness and Sensitivity

SAOE is a very sensitive ML material and the green light emitted can be observed by naked eyes when gently scratched in a weak light environment. In the elastic range, the ML is approximately linearly proportional to the applied dynamic force/pressure, and the ML intensity or brightness will linearly increase as the external stress/strain is increased.^[^ ^16^ ^]^ The matrix intrinsically has weak or no ML properties and requires physical and chemical modulation to make it luminescent and to enhance its luminescence. The ML intensity/brightness and sensitivity of SAO‐based phosphors are mainly enhanced by the following three strategies: I) codoping with different ions,^[^ ^13^ ^]^ II) optimizing the powder synthesis process,^[^ ^22^, ^23^, ^24^ ^]^ and III) developing and selecting different composite processes for powders and substrates.^[^ ^25^ ^]^

Among these, greater enhancement in ML is achieved through the codoping of luminescent ions. It has been found that doping with Eu ions can provide luminescent activators that finally convert mechanical energy into visible light and—to some extent—can greatly improve the observed ML brightness. It is noted that SAOE has instrument‐recognizable ML. Codoping with Dy^3+^ ions can provide additional trap levels at the proper depths to further increase ML brightness and extend the persistence of the afterglow. Zr^4+^ ions can moderate the microstructure and create new electronic states in the crystal to improve ML brightness and simultaneously increase the sensitivity of SAOE/SAOED to tiny stress applied.^[^ ^26^, ^27^, ^28^ ^]^ B or P ions introduced through boric acid or ammonium dihydrogen phosphate, respectively, as flux and structural modifiers enable afterglow enhancement by increasing the trap depths.^[^ ^13^ ^]^

Powder synthesis processes can be optimized by proper selection of calcination process parameters and precursors. The most commonly employed calcination technique is the solid‐state reaction method (SSRM), while the combustion method (CM) is also sometimes adopted. SSRM allows precise and easier control over annealing duration, annealing temperature, annealing atmosphere and other aspects to obtain desired products. Inorganic oxides are usually chosen as the precursors for the SSRM. Since chlorides normally have lower melting points, they are used as flux and dopants are commonly introduced by corresponding chlorides (e.g., Dy^3+^ doping through the introduction of DyCl_3_ rather than Dy_2_O_3_). Further, the proper introduction of chloride ions facilitates formation of new SAO structures,^[^ ^29^, ^30^ ^]^ leading to heterostructural or interfacial effect, which will improve the luminous intensity and brightness of SAO.^[^ ^31^, ^32^, ^33^, ^34^ ^]^ Additionally, boric acid is frequently employed as flux to boost the melting effect.^[^ ^35^ ^]^

In order to obtain an operable device against external force stimuli, the prepared SAO ceramic powders are usually composited with or incorporated to a deformable substrate/matrix such as epoxy resin^[^ ^5^ ^]^ or PDMS,^[^ ^36^ ^]^ and then directly coated/painted onto a substrate to create a sensor. Improvements on the compositing process via the use of ultrasound,^[^ ^25^ ^]^ high‐temperature spray techniques,^[^ ^37^ ^]^ and radio‐frequency (RF) sputtering techniques^[^ ^38^ ^]^ also impact significantly on the ML performance of the final composite.

#### 3.1.1 Ion Doping

SAO can be moderated by varying the ratio of Eu^2+^, Dy^3+^, and Nd^3+^ dopants to selectively achieve stress‐free persistent PL (afterglow) or ML. Appropriate concentrations of Eu^2+^, Dy^3+^, and H_3_BO_3_ can result in good initial ML brightness and a linear relationship between the force/pressure and the luminous intensity. However, these “appropriate” concentrations vary with the sample preparation methods, which include the calcination temperature, the duration of calcination, and the calcination atmosphere (e.g., the proportion of H_2_ in the gas mixture). In addition, ML property of SAOE is more sensitive to experimental conditions than SAOED; the former has a relatively low stress‐free afterglow, whereas the latter has brighter ML but shows strong inherent long afterglow properties.^[^ ^39^ ^]^ There is no significance difference between the ML intensities of SAOE and SAOEN(Eu^2+^, Nd^3+^), but the latter has better ML linearity.^[^ ^24^ ^]^ Finally, a stress‐free afterglow and ML intensity and a stress‐induced afterglow are complementary in SAOED. In some application scenarios such as fast‐response dynamic stress/strain sensors, where the stress‐free afterglow needs to be suppressed, the functionality could be achieved by regulating the concentrations of codoped lanthanide ions, such as Dy^3+^ dopants.

The codoping of Eu^2+^ with Dy^3+^ or Nd^3+^ to enhance the afterglow of SAOE was first proposed by Matsuzawa et al. in 1996.^[^ ^4^ ^]^ This enhancement in the afterglow can be credited to the formation of a high density of trap levels by the Dy^3+^ ions at suitable depths in the SAO crystal lattice, which ensures bright and long‐lasting phosphorescence at room temperature. In their experiments, a concentration of 1% Eu ions is fixed, while the doping concentrations of Dy^3+^ and Nd^3+^ are rationally adjusted. And, the experiments show that the optimum concentrations of Dy^3+^ and Nd^3+^ are 2% and 1%, respectively. Under the synergistic effect of doping and metrology, an obvious improvement in the PL of the material was achieved. It is speculated that the differences in the optimal doping concentration of different ions result from the different solubilities of the two ion types in the system (i.e., the ionic radius of Dy^3+^ is slightly smaller than that of Nd^3+^, so it may be more soluble in the matrix). In addition, adequate Dy^3+^ doping concentration can facilitate formation of more trap levels compared to doping with Nd^3+^ ions, resulting in brighter phosphorescence. However, the byproducts DyAlO_3_ or NdAlO_3_ are produced when the dopants exceed their solubility limits,^[^ ^4^ ^]^ which will weaken the intensity.

**Figure 5:**
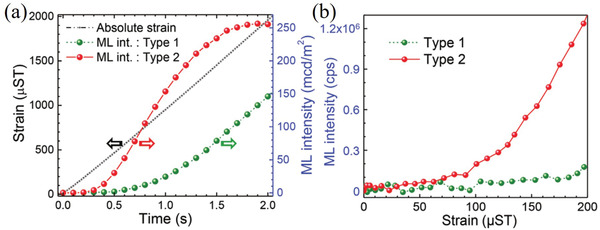
a) ML of two types of sensors detected by a charge‐coupled device (CCD) camera compared to the simultaneous absolute strain and loading time. b) ML of two types of sensors at small strains when detected by a photon counter. Reproduced with permission.^[^ ^27^ ^]^ Copyright 2018, Wiley‐VCH.

After optimization and a series of studies on the ML of SAO‐based materials by Xu and co‐workers, Kim et al. proposed that Dy^3+^ and Nd^3+^ ions could enhance the PL persistence and improve the ML brightness of SAOE. In their experiments, they concluded that the optimum concentrations of Eu^2+^, Dy^3+^, and Nd^3+^ ions are 1% for each (in 75% N_2_ and 25% H_2_ atmosphere).^[^ ^40^ ^]^ Lin et al. from Tsing Hua University proposed that Dy^3+^ doping concentration has an evident effect on the ML intensity, while Eu^2+^ doping concentration within a range of 0.5–1.5 mol% has less effect on the ML intensity.^[^ ^41^ ^]^ Three samples with different Dy^3+^ codoping concentrations (1%, 2%, and 3%) were prepared. All the prepared samples were excited with UV light (365 nm) for 5 min and then stored in the dark for ≈30 h to eliminate the afterglow. Then, 500‐g spheres were dropped from a height of 20 cm to excite the ML. A Dy^3+^ doping concentration of 3% would lead to a decrease in the ML intensity, which is ascribed to the recapture of free holes released after applying a stress by excessive number of traps, resulting in ML quenching. Tu and co‐workers improved the ML brightness and strain sensitivity of SAOE by introducing Zr ions to generate new electronic states in crystal.^[^ ^26^ ^]^ The performance of ML sensors based on the prepared samples showed substantial improvements from that of previous sensors (**Figure** **5**a,b).^[^ ^27^ ^]^

**Figure 6:**
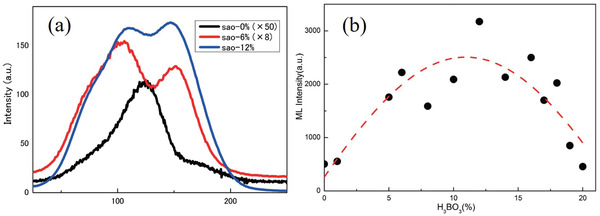
a) TL intensity versus temperature (for comparison, the intensities for 0 and 6 mol% were multiplied by factors 50 and 8, respectively). b) ML intensity versus H_3_BO_3_ concentration. Reproduced with permission.^[^ ^35^ ^]^ Copyright 2017, The Ceramic Society of Japan.

Boric acid is a typical fluxing agent that improves the efficiency of the solid‐state reaction by enabling lower annealing temperatures, and it promotes the entry of dopants and their uniform dispersion in the lattice to form luminescence centers. In addition, the introduced B^3+^ can increase the trap depths and improve the ML and PL of SAOE to a certain degree.^[^ ^13^ ^]^ The optimal concentrations of dopants vary with different sample preparation methods. Experiments show that the optimal amount of boric acid added during the preparation of SAOE by the combustion method is 7.5%,^[^ ^42^ ^]^ whereas the optimal amount is 12% for the solid‐phase method^[^ ^35^ ^]^ (**Figure** **6**a,b).

This difference may be related to the size difference of the raw material particles and the fluctuations in the milling time and other experimental parameters. In addition, the use of boric acid leads to the aggregation of SAOE particles, hence an increase in particle size. Interestingly, the addition of H_3_BO_3_ enhances ML but decreases PL for SAOE fired in a reducing atmosphere (95% Ar + 5% H_2_) at 1500 °C for 8 h using the solid‐phase method. The enhancement in the ML intensity reached a maximum of 2.5 times with the addition of 12% H_3_BO_3_,^[^ ^35^ ^]^ but the PL decreased by more than two times. The trap concentrations determined from TL measurements suggest that the ML enhancement results from an increase in the concentration of traps released by mechanical stimuli, while the stronger self‐absorption of the sample causes weakening of PL. Simultaneous improvement in the intensities of ML and PL will be of great significance for various applications of SAO phosphors.

**Figure 7:**
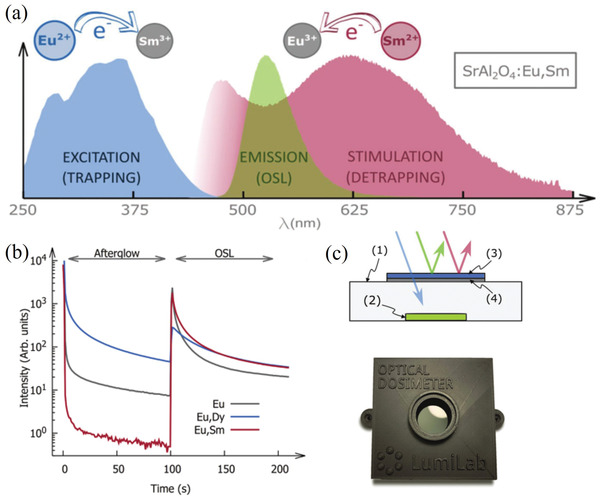
a) Absorption of light with wavelengths of 250–475 nm by SrAl_2_O_4_:Eu^2+^, Sm^3+^, which is stored in the deep Sm^3+^ traps (blue curve). Detrapping occurs when illuminated with light having a wavelength of 500–850 nm (red curve), producing light emission and a long afterglow with wavelengths of 475–625 nm (green curve). b) Comparison of the afterglow and optically stimulated luminescence (OSL) responses of SrAl_2_O_4_:Eu^2+^; SrAl_2_O_4_:Eu^2+^, Dy^3+^; and SrAl_2_O_4_:Eu^2+^, Sm^3+^. Excitation by a violet laser (*λ* = 405 nm) was stopped at *t* = 0 s, and the three materials exhibited different afterglows: SrAl_2_O_4_:Eu^2+^, Dy^3+^ was the strongest, SrAl_2_O_4_:Eu^2+^ second, and SrAl_2_O_4_:Eu^2+^, Sm^3+^ had almost no afterglow. At *t* = 100 s, an infrared laser (*λ* = 808 nm) was used to continuously excite the three materials. SrAl_2_O_4_:Eu^2+^, Sm^3+^ had the highest OSL intensity, which indicated that the carriers stored in the Sm^3+^ traps began to be released. c) Conceptual diagram and principles of the light sensor used to measure daylight intensity. Blue light from the sunlight can pass through a shortpass or bandpass filter (3) and be absorbed by the polymer layer containing SrAl_2_O_4_:Eu^2+^, Sm^3+^ (2) underneath the neutral density filter (4) and an opaque shell (1). Reproduced with permission.^[^ ^43^ ^]^ Copyright 2021, Wiley‐VCH.

It is noted that the PL afterglow of a sample will be significantly prolonged after the addition of boric acid, which intermingles with the ML of SAOE itself to some extent, resulting in subtle ML to naked eyes. The presence of B^3+^ promotes a more uniform distribution of Eu^2+^ in the sublattice of Sr^2+^, which accomplishes one of the necessary conditions for a long afterglow.^[^ ^13^ ^]^ We know that SAOED exhibits a long afterglow due to deep traps. Deeper trap defects can be produced by replacing Dy^3+^ with Sm^3+^. Such deep traps cannot be spontaneously eliminated at room temperature, but they can be eliminated by stimulating with near‐infrared (NIR) light, resulting in green PL and afterglow (**Figure** **7**a,b). Van der Heggen et al. found that codoping of Eu^2+^ and Sm^3+^ results in trap filling and detrapping after irradiation with different wavelengths of light, which is beneficial for optical sensors (Figure 7c),^[^ ^43^ ^]^ where the ML phenomenon was not reported. When irradiated with blue‐violet or UV light, electrons transfer from Eu^2+^ to Sm^3+^, and the material glows green; however, the afterglow is much weaker than that for SAOED samples owing to the deep traps. When irradiated with NIR light, electrons transfer from Sm^2+^ to Eu^3+^, the material still glows green, and the afterglow is similar as SAOED.

#### 3.1.2 Use of Nitrate Decomposition

Xu and co‐workers found that SAOE prepared using nitrate decomposition had a higher sensitivity to small stresses compared to that prepared by the solid‐phase method, probably because SAOE decomposed from nitrate had shallower trap levels, larger particle sizes, and a slab‐like shape.^[^ ^22^ ^]^

In a typical solid‐phase method, SrCO_3_, *α*‐Al_2_O_3_, and Eu_2_O_3_ were precisely weighed to a stoichiometric molar ratio and continuously milled by zirconia balls in ethanol for several hours. The SAOE precursor was obtained by drying the resulting suspension. In the nitrate decomposition method, the raw materials, metal nitrates Sr(NO_3_)_2_, Eu(NO_3_)_3_·6H_2_O, and Al(NO_3_)_3_·9H_2_O were dissolved in distilled water, and the aqueous solution was thoroughly mixed by ultrasound for ≈1 h. The SAOE precursors were then vigorously stirred on a hot plate and evaporated until dry in the atmosphere. Precursors obtained by the above two methods were calcined at 800 °C in air for 1 h, followed by sintering at 1350 °C in a 5% H_2_/95% Ar atmosphere for 4 h to obtain SAOE powders. The SAOE powders synthesized by the solid‐phase reaction method and nitrate decomposition method are denoted as SS‐SAOE and ND‐SAOE, respectively. Then, the SAOE powders were mixed with an epoxy resin and made into ML films by screen printing for testing.

**Figure 8:**
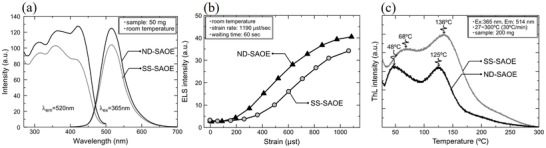
a) PL spectra, b) ML response curves, and c) TL curves obtained at a heating rate of 30 °C min^−1^ for SS‐SAOE and ND‐SAOE. The abbreviation ELS stands for elasticoluminescence. Reproduced with permission.^[^ ^22^ ^]^ Copyright 2014, World Scientific Publishing Co Pte Ltd.

ND‐SAOE is generally more sensitive than SS‐SAOE (**Figure** **8**a,b). Scanning electron microscopy (SEM) characterization revealed that the spherical SS‐SAOE particles agglomerate to sub‐micrometer‐sized. ND‐SAOE particles agglomerate to several micrometers, which have a plate‐like shape with clearly visible grain boundaries between particles. The two SAOE powders were sintered at the same temperature but have different particle sizes and microscopic morphologies, leading to different sensitivities of ND‐SAOE to minor stresses. The precursor powders prepared by nitrate decomposition is nanosized, so have a large specific surface area, which can fully withstand a mechanical force to achieve effective light emission. By contrast, deformation along a crystal plane in a special orientation may be more sensitive to stress owing to the anisotropy of the crystal.

In addition, the carriers captured by the two types of SAOE were analyzed using TL glow curves. The heating rate was fixed at 30 °C min^−1^ for all measurements. The measurements showed that the TL glow curves of each of the two SAOE powders had two peaks (Figure 8c). The peaks of SS‐SAOE locates at 68 and 136 °C, and those of ND‐SAOE locate at 48 and 125 °C. ND‐SAOE has shallower trap levels than SS‐SAOE. These results lead to a conclusion that the carriers trapped in defects in ND‐SAOE are more easily released by relatively smaller thermal and mechanical energies compared to those in SS‐SAOE.

#### 3.1.3 Matrix and Process Selection

When developing dynamic stress/strain and pressure sensors using SAO, it is often necessary to incorporate the powders into deformable matrices, and the selection of a proper processing method is also beneficial for sensor performance improvement.

Epoxy resin is a thermoset resin (harder) with a good adhesive strength and chemical resistance to a variety of materials. PDMS (softer) is a hydrophobic silicone that is chemically stable, flexible, flowable and has a film‐forming ability.^[^ ^44^ ^]^ Both of them are most commonly used as matrices or substrates for compounding with SAO owing to their low cost and flexibility. After lamination, the materials can be cured by direct deposition, screen printing or squeegee methods to form ML sensors with specific shapes such as films, sheets, spheres, disks, and dog bone‐shape to meet practical application requirements.^[^ ^33^, ^34^, ^35^, ^36^, ^37^, ^38^, ^39^, ^40^, ^41^, ^45^, ^46^, ^47^, ^48^, ^49^, ^50^, ^51^, ^52^ ^]^

**Figure 9:**
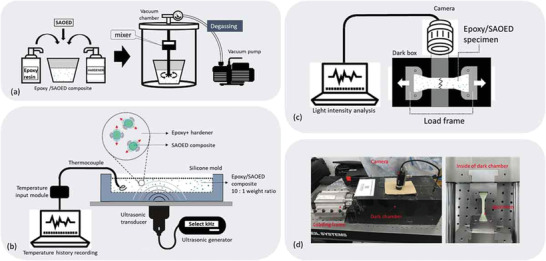
a) Preparation of the composite and evacuation/degassing process. b) Application of ultrasonic waves during the curing process. c) Schematic of the test setup for the ML sensitivity and d) real test setup. Reproduced with permission.^[^ ^25^ ^]^ Copyright 2020, Taylor and Franices.

**Figure 10:**
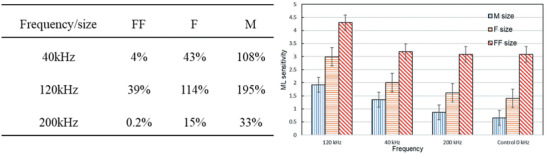
Comparison of the ML improvement and sensitivity for different sizes of particles and exposure to ultrasound at different frequencies. Reproduced with permission.^[^ ^25^ ^]^ Copyright 2020, Taylor and Franices.

Yun and co‐workers at Seoul National University found that ultrasonic vibration applied during the hardening stage can help improve the bonding between SAOED particles and epoxy resin when mixing (**Figure** **9**a–d), so that the ML sensitivity of the composite can be significantly improved without changing the PL (**Figure** **10**). However, this effect has only been studied for epoxy resin, and the effect for other mixed materials is yet to be studied.^[^ ^25^ ^]^ For a smaller particle size, the force transfer from epoxy to the ML particles will be more effective, so the sensitivity will be higher. Three sizes of SAOED particles, 27.09 (FF size), 15.46 (F size), and 3.328 (M size) µm, were selected for investigation, and ultrasound sonication at frequencies of 200, 120, and 40 kHz was applied during the curing process. It was found that the ML sensitivities of all composites were improved to different degrees, which can be deduced from the table in Figure 10. When ultrasound is applied, the dependency of ML on crystal size is less than those without ultrasound. In general, the highest ML sensitivity was obtained for the combination of FF particles sonicated at a frequency of 120 Hz.

**Figure 11:**
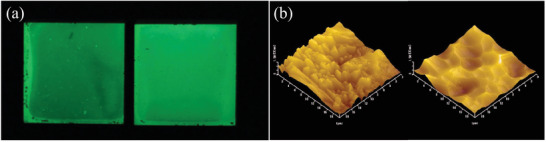
a) Picture of SAOE/Al_2_O_3_/Si films prepared by the bilayer method taken after 10 min of irradiation by a UV lamp. Reproduced with permission.^[^ ^55^ ^]^ Copyright 2013, Elsevier B.V. b) 3D surface topography of the Al plate (left) and SAOE thin film (right) prepared by the RF sputtering method, as measured by atomic force microscopy (AFM). Reproduced with permission.^[^ ^15^ ^]^ Copyright 2007, The Electrochemical Society.

The performance of ML sensors made of composite materials varies with the stress transfer capability of the flexible material to the SAOE particles, and the total ML sensitivity is reduced owing to the mixing with other nonmechanoluminescent materials. Therefore, to overcome this shortcoming, ML particlescan be directly deposited onto substrates such as Al plates,^[^ ^15^ ^]^ alloys,^[^ ^37^ ^]^ quartz glass,^[^ ^38^ ^]^ and other materials with good adhesion and crystallinity by physical vapor deposition^[^ ^53^ ^]^ and RF sputtering techniques (**Figure** **11**b)^[^ ^54^ ^]^ or onto ceramic sheets by high‐temperature fusion techniques.^[^ ^37^, ^52^ ^]^

Fu et al. proposed a bilayer method to prepare SAOE thin filmss. These films have a four‐layer structure, with the bottom layer being the silicon substrate, the middle layer being a 200‐nm‐thick Al_2_O_3_ heterogeneous buffer layer to eliminate the large lattice difference and thermal mismatch between SAOE and the silicon substrate, the upper layer being a 600‐nm‐thick SAOE homogeneous buffer layer to reduce the internal stress during film growth, and the top layer being a 1.5‐µm‐thick SAOE film deposited by continuous sputtering method. Thick SAOE/Al_2_O_3_/Si films obtained by this method have excellent PL and ML properties (Figure 11a).^[^ ^55^ ^]^

**Figure 12:**
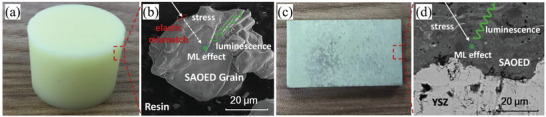
Samples of conventional resin‐based ML sensors and newly developed ML ceramic sensors. a) Conventional ML sensors doped with SAOED in epoxy resin and b) their microstructure. c) Laboratory‐prepared ultrasensitive ML ceramic sensors and d) their microstructure. Reproduced with permission.^[^ ^37^ ^]^ Copyright 2020, Elsevier B.V.

Cai et al. reported an ultrasensitive ML sensor prepared by air plasma spraying (APS) technique. The sensor has a three‐layer structure (**Figure** **12**a–d). The bottom substrate is 3‐mm‐thick Hastelloy. The middle layer is yttrium‐oxide‐stabilized zirconia (YSZ) with a high fracture strength and toughness, being sprayed onto the substrate (thickness ≈50 µm) via the APS to improve the adhesion between SAOED and the substrate. The top layer is an aluminate directly deposited onto YSZ (thickness ≈30–40 µm) by melting at an ultrahigh temperature of 3000 °C.

In this scheme, the ultrahigh process temperature led to the deformation or even destruction of the lattice of the ML material, so after the sensor is fabricated, vacuum tempering is required to reduce the lattice distortion and to restore phosphorescent properties. The final product has a high sensitivity (kilopascal scale) and a low sensitivity threshold due to the absence of a support substrate.^[^ ^37^ ^]^

### 3.2 Color during ML/Spectral Tuning

SAOE powder itself appears light green under white light, and both its ML and PL are green with a center wavelength of 520 nm, which is presently the main research direction. However, it can be synergistic with other ions and emit different colors/spectra during ML (**Table** **1**).

**Table 1:** Typical host‐dopant combinations for making ML phosphors^[^ ^56^ ^]^

Host	Dopant	ML [nm]	PL [nm]	EL [nm]	Refs.
SrAl_2_O_4_	Eu^2+^, Eu^2+^/Dy^3+^	524	524	524	[5, 6, 40]
	Ce^3+^, Ce^3+^/Ho^3+^	300–500	300–500	–	[57]
	Eu^2+^/Er^3+^	524, 1530	524, 1530	–	[58]
	Eu^2+^, Cr^3+^, Nd^3+^	750–1000	520, 900–1150	–	[24]

**Figure 13:**
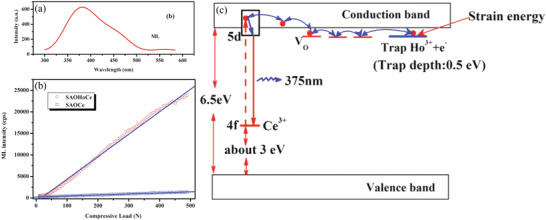
a) ML spectrum of SAO:Ce^3+^, Ho^2+^, b) linear ML relationship, and c) energy‐level relationship during the ML process. Reproduced with permission.^[^ ^57^ ^]^ Copyright 2007, American Institute of Physics.

Xu's team achieved UV ML near 375 nm by doping SAO with Ce^3+^ and Ho^2+^.^[^ ^57^ ^]^ Among them, Ce^3+^ mostly substitutes Sr^2+^ because their ionic radiuses are similar, and Ce^3+^ follows the allowed transitions of 4f–5d (**Figure** **13**a–c). The fraction of electron energy of Ce^3+^ in the 5d state is higher than the CB of the host, and electrons may leave the CB by thermal excitation or thermally assisted photoexcitation. When Ce^3+^ in SAO:Ce^3+^, Ho^2+^ is excited by UV light with a wavelength of 254 nm, ground‐state electrons transition from 4f to 5d, where the excited electrons partially located in the CB will escape under thermal perturbation or quantum tunneling. Subsequently, these electrons are captured by O holes and move from one trap to another under external stimuli such as thermal or mechanical energy. Since Ho^2+^ is located in a 5d state particularly close to the CB of SAO host, electrons can be readily captured, and these electrons can be excited by stress stimulation. In addition, these electrons can rapidly relax through O traps and the CB nonradiatively back to the lowest 5d state in a Ce^3+^ ion and then return to the ground state by emitting UV light with a wavelength of 375 nm.

**Figure 14:**
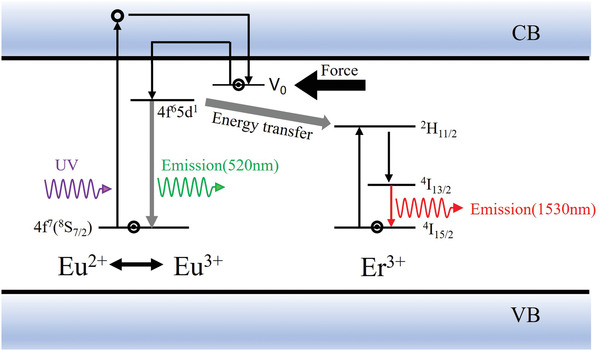
NIR ML mechanism of SAO:Eu, Er. Reproduced with permission.^[^ ^59^ ^]^ Copyright 2011, The Electrochemical Society.

ML in the NIR region is achieved by codoping with Eu^2+^ and Er^3+^ (**Figure** **14**), although the light intensity remains very weak.^[^ ^58^ ^]^ Luminescence in the NIR region is sometimes designed by the downshifting, downconversion, and quantum cutting effects, which cause the resonance energy to move from a donor energy level to an acceptor energy level. Specifically, a sender ion absorbs visible‐light photons; then, an acceptor ion emits NIR photons. A Eu^2+^ electron in the ^8^S_7/2_ state is subsequently trapped owing to UV excitation, and this electron is released when stimulated by stress to recombine with the Eu^3+^ to produce Eu^2+^ in an excited state (4f^6^5d^1^). Some of these Eu^2+^ ions will transfer to the ground state and emit photons with a wavelength of 520 nm, while others will transfer energy to the Er^3+^ ions (^2^H_11/2_), followed by a nonradiative transition from ^2^H_11/2_ to ^4^I_13/2_, and then, from ^4^I_13/2_ to ^4^I_15/2_ to emit infrared light.

**Figure 15:**
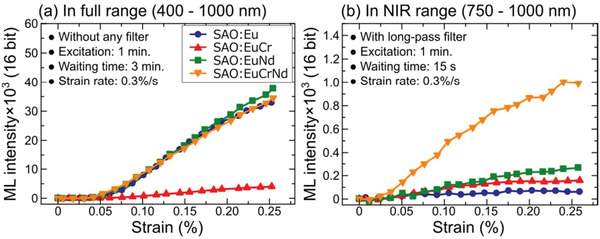
ML intensity versus strain for SAO:Eu; SAO:Eu, Cr; SAO:Eu, Nd; and SAO:Eu, Cr, Nd in the a) 400–1000 and b) 750–1000 nm bands. Reproduced with permission.^[^ ^60^ ^]^ Copyright 2021, The Electrochemical Society.

**Figure 16:**
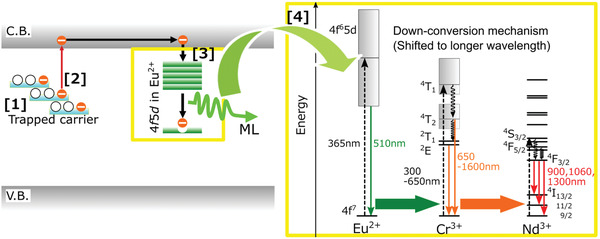
Schematic of the ML and energy transfer (downconversion) mechanisms in SAO:Eu, Cr, Nd. Reproduced with permission.^[^ ^60^ ^]^ Copyright 2021, The Electrochemical Society.

Stronger NIR ML could be achieved by codoping with Eu^2+^, Cr^3+^, and Nd^3+^ (**Figure** **15**), and nitrates were used as precursors in experiments and assisted by organic acids to achieve strong NIR luminescence and a total integral ML intensity almost identical to SAOE in the 400–1000 nm band.^[^ ^60^ ^]^ Subsequently, through PL measurements and analysis, it was proposed that this NIR light originates from the successive transfer of trapped carriers to Cr^3+^ and Nd^3+^ during the process of being released to the transition state in Eu^2+^ under stress (**Figure** **16**). Further, Cr^3+^ acts as a sensitizer during this energy transfer process for amplification and relies on Nd^3+^ to harvest energy and finally emit light via ML in the 750–1000 nm band.

### 3.3 Improving Water Proofing

SAO has poor water resistance. Typically, the ML will fail after submersion in water for a few days, which is extremely unfavorable. An aluminate‐based persistent phosphor was first introduced to road striping paint by the OssN239 road in the Netherlands to give the road markers a beautiful self‐luminous effect at night. However, the road markers lost their fluorescent function in less than 14 days owing to rainfall.^[^ ^61^ ^]^ A study demonstrated a reduction in fluorescent brightness when SAO absorbs more than 3% water,^[^ ^62^ ^]^ and the following hydrolysis reaction occurs^[^ ^56^ ^]^

(1) $${\mathrm{SrAl}}_{2}O_{4}+4H_{2}O\to {\mathrm{Sr}}^{2+}+2{\mathrm{OH}}^{-}+2\mathrm{Al}{\left(\mathrm{OH}\right)}_{3}↓$$

The problem of being not waterproof greatly limits SAO's application because it is unavoidable in most cases that SAO will be exposed to humid air for a long duration or accidentally contact water. To solve this problem, coating the surfaces of the SAO particles with a water‐resistant substance has been proposed. Since SAO is a material with a long afterglow, existing waterproofing solutions are more mature, and the materials are more abundant, including oxide metal shells, alkyl phosphates, organic polymers, and aerogels.^[^ ^63^, ^64^ ^]^ However, there are fewer waterproofing solutions for SAO when it is used as an ML material. Currently epoxy resin and PDMS are used for coating because they have good mechanical deformation and elastic characteristics while taking into account waterproofing. It has been verified that SAO and epoxy resin or PDMS composites can protect the material in 60 °C hot water for up to 1 week. The duration of protection can reach six months or even two years^[^ ^65^ ^]^ in the outdoor open‐air conditions for bridge pressure monitoring.

## 4 Luminous Properties and Crystal Structure

### 4.1 Photoluminescence

**Figure 17:**
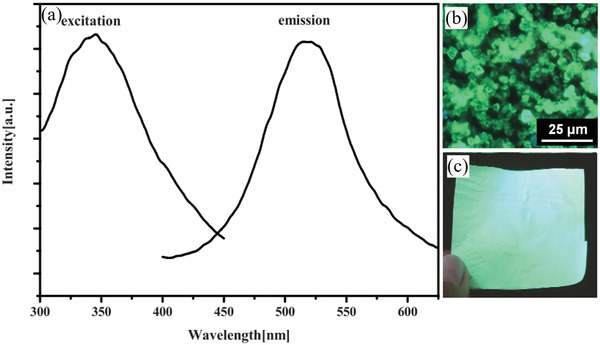
a) PL spectra of SAOE films. Reproduced with permission.^[^ ^66^ ^]^ Copyright 2009, The Electrochemical Society. b) Fluorescence microscopy image of SAOE particles. c) SAOE sheet sensor. Reproduced with permission.^[^ ^67^ ^]^ Copyright 2012, SPIE.

Light with wavelengths of 250–500 nm can excite SAOE to emit light (**Figure** **17**a). However, the intensity of the emission depends on the wavelength of excitation, and the strongest excitation is at 365 nm. Light‐emitting diodes (LEDs) and sunlight under natural conditions can be partially absorbed to excite emission, and the center wavelength for broad emission is near 520 nm, which is green light and extremely obvious to naked eyes (Figure 17b,c).

**Figure 18:**
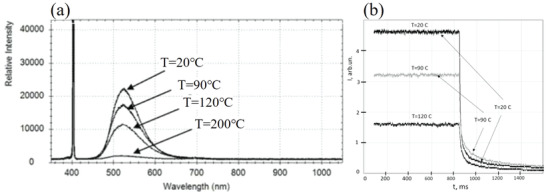
a) PL spectra and b) photoluminescent afterglow of SAOED at different temperatures. Reproduced with permission.^[^ ^68^ ^]^ Copyright 2020, IOP Publishing Ltd.

Like most luminescent materials, the luminous intensity of SAO has an inverse relation with temperature.^[^ ^69^, ^70^, ^71^ ^]^ Banishev and Banishev confirmed a higher temperature results in weaker PL for SAOED (**Figure** **18**a). Temperature also affects the photoluminescent afterglow, and the order of the photoluminescent afterglow intensity was measured as 90 °C > 20 °C > 120 °C (Figure 18b), indicating that excessive heat will lead to a thermal burst of luminescence.^[^ ^68^ ^]^ Several studies have also demonstrated that temperature changes the spectral properties of other ML materials;^[^ ^72^, ^73^, ^74^, ^75^ ^]^ some focus on the temperature characteristics of ML, while most focus on the temperature quenching characteristics.

### 4.2 Afterglow

**Figure 19:**
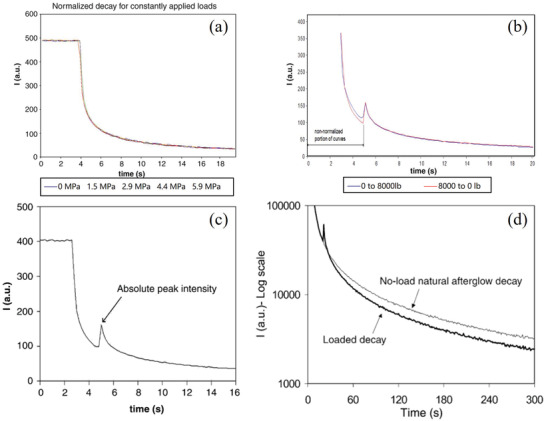
a) Decay curves of the afterglow for different static stresses. b) Decay curves of the afterglow during loading and unloading stresses. c) Luminescence caused by applying an 8 kPa load during the afterglow. d) Comparison of decay curves during the afterglow with no load and an applied 8 kPa load. Reproduced with permission.^[^ ^11^ ^]^ Copyright 2005, Elsevier B.V.

SAOE itself is a persistent phosphor with a typical long afterglow. The PL of SAOE (SrAl_2_O_4_:Eu) basically has no obvious afterglow, while SAOED (SrAl_2_O_4_:Eu/Dy) has a longer photoluminescent afterglow and much longer force‐induced luminescent afterglow compared to ZnS:Mn, mainly because the carriers (holes) in its traps can be excited at room temperature. If the stress/strain is static, the decay process of the afterglow will not change (**Figure** **19**a). In contrast, dynamic loading, whether loaded or unloaded, will excite ML and impact on the subsequent afterglow process (Figure 19b–d).^[^ ^11^ ^]^

**Figure 20:**
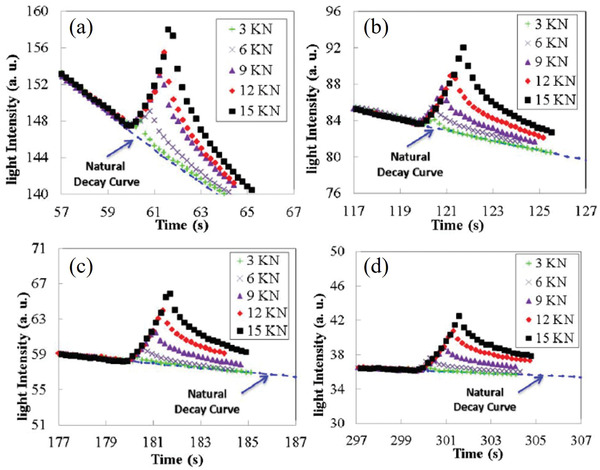
Stress‐state PL decay curves for different static stresses at a strain rate of 0.3 mm s^−1^: decay times of a) 1, b) 2, c) 3, and d) 5 min. The blue dashed lines indicate the no‐stress PL decay. Reproduced with permission.^[^ ^76^ ^]^ Copyright 2014, Acta Materialia Inc. Published by Elsevier Ltd.

Yun and co‐workers explored the change in the afterglow of SAOED at a certain strain rate, and the decay rate of the ML afterglow of SAOED is much faster than that of the unstressed afterglow, although its intensity eventually decays to a level similar to that of the unstressed afterglow (**Figure** **20**a–d). It is noted that doping with Dy^3+^ will have a more obvious force‐induced luminescent afterglow, hence, the material chosen for their experiment was SAOED instead of SAOE.^[^ ^76^ ^]^

**Figure 21:**
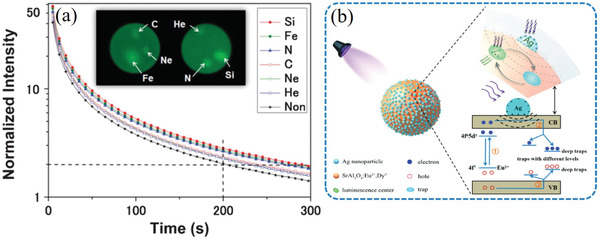
a) Afterglow decay curves for SAOE samples that are un‐irradiated and have regions bombarded with different heavy ions. Reproduced with permission.^[^ ^79^ ^]^ Copyright 2012, The Royal Society of Chemistry. b) Diagram showing the mechanisms of the Ag‐enhanced afterglow properties. The AgNPs are uniformly distributed on the surface of SAOED to form an electrostatic field, which can assist in the formation of more photogenerated electrons captured by traps at different depths under illumination, resulting in a longer afterglow after the illumination stops. Reproduced with permission.^[^ ^80^ ^]^ Copyright 2022, The Royal Society of Chemistry.

In addition, if we disregard the ML properties of SAOE and only aim to enhance the stress‐free afterglow aspect of SAOE, there are methods such as heavy‐ion bombardment (**Figure** **21**a) and the deposition of Ag nanoparticles (NPs) on the surface of SAOE (Figure 21b). Moreover, we can adjust the wavelengths of afterglow by doping with different ions and using fluorescent dyes.^[^ ^77^, ^78^ ^]^ SrAl_2_O_4_:Eu^2+^, Ho^3+^ shows a great enhancement in its afterglow after bombardment with different heavy ions, which may be due to strong electron‐blocking instincts. In subsequent analyses using X‐ray diffraction, TL and afterglow curves, it was found that heavy‐ion bombardment did not change the crystal structure of sample nor increase the trap depth but only increased the density of traps, similar to the original traps. Heavy‐ion bombardment in its incident trajectory produces energy several orders of magnitude greater than conventional ionizing radiation, and the resulting dense electron excitation creates more lattice defects along the trajectory, thus enhancing the intensity of the afterglow.^[^ ^79^ ^]^

**Figure 22:**
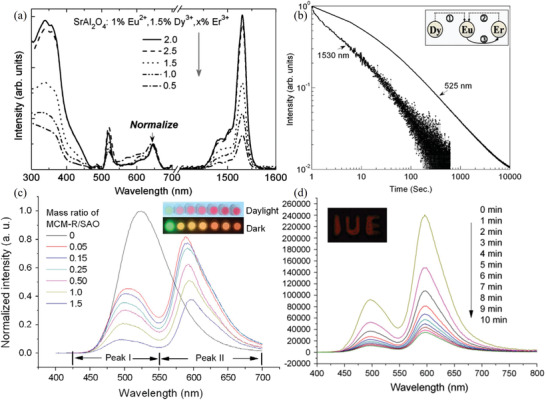
a) NIR emission spectra of SrAl_2_O_4_:Eu^2+^, Dy^3+^, Er^3+^ at different Er^3+^ doping concentrations (ex = 340 nm). b) Afterglow curves of SrAl_2_O_4_:1.0% Eu^2+^, 1.5% Dy^3+^, and 2% Er^3+^ at 525 and 1530 nm. The inset shows the energy transfer process between Eu, Dy, and Er ions. Reproduced with permission.^[^ ^77^ ^]^ Copyright 2009, AIP Publishing LLC. c) Emission spectra of rhodamine MCM‐R/SAO mixtures after mixing at different mass ratios. d) Afterglow decay of the rhodamine MCM‐R/SAO mixture mixed at 1:1 (mass ratio). The inset shows a fluorescence image after compounding the mixture with epoxy resin. Reproduced with permission.^[^ ^78^ ^]^ Copyright 2012, Elsevier B.V.

Hai et al. found that the deposition of AgNP on the surface of SAOED doubled the intensity of the afterglow during decay. AgNP can induce an electric field on the surface of SAOED, promoting the separation and transfer of photogenerated electrons and holes and the capture of more photogenerated electrons by deep traps, thus increasing the intensity of the luminescence during the slow decay process. In addition, this method requires a 15% SiO_2_ surface coating in order to protect the AgNP on the SAOED surface.^[^ ^80^ ^]^ Yu et al. codoped SAO with Eu^2+^, Dy^3+^, and Er^3+^ to achieve a continuous afterglow in the NIR band (peak at 1530 nm) for more than 10 min (**Figure** **22**a,b).^[^ ^77^ ^]^ Zhang and co‐workers extended the fluorescence band of SAOED to 550–700 nm band (peak at 600 nm) by encapsulating rhodamine B in mesoporous silica nanoparticles (MCM‐R) as a color conversion agent (Figure 22c,d).^[^ ^78^ ^]^

### 4.3 AC Electroluminescence

**Figure 23:**
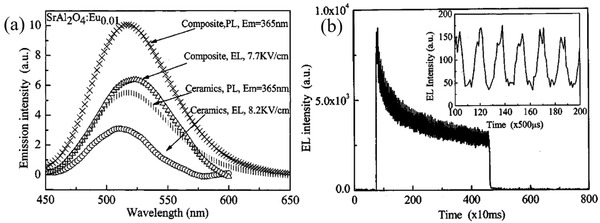
a) ML and PL of two SAOE spheres, one of which was directly prepared by RF sputtering (SAO‐E ceramics), and the other sphere is processed by sputtering, mixing and pressing of SAOE and fluoropolymer (commonly used in the preparation of coatings). b) AC EL response of SAO‐E ceramics. The inset shows the details of the modulated photoemission signal. Reproduced with permission.^[^ ^81^ ^]^ Copyright 2004, American Institute of Physics.

In the SAO system, there is an interdependent relationship between force, light, and electricity. During material deformation, two opposite surfaces will appear to have positive and negative charge due to the mechanical force acting on them, and an internal polarization phenomenon occurs, which is the “piezoelectric effect.” A force generates an electric field by the piezoelectric effect, so the carriers in trap centers are released, and the luminous center compounds become luminous. It has been confirmed that SAOE could emit intense green light when excited by lower direct current (DC) or alternating current (AC) voltages (**Figure** **23**a,b).

Usually, ML, PL and AC‐EL are all derived from doping ions in the matrix, and their spectral peak positions are consistent. The main peak positions of ML/AC‐EL and PL may have a bit offset, mainly because PL test of a sample is in a static condition, while ML/AC‐EL test with a dynamic loading, thus there are subtle changes in ion spacing and crystal field, and may also be affected by factors such as the types of defects in the matrix. Without considering the thermal effect, the PL of the strontium aluminate can sustain without weakening as long as it is continuously irradiated with ultraviolet light, whereas the repeatability of ML and EL decays with the cycling of the applied force/electric field. Nevertheless, Like most other inorganic ML materials, strontium aluminate has thermal excitation luminescence. Almost all doped luminescent powders have thermoluminescence and show bright thermally stimulated luminescence when scalded with hot water, which is mainly due to the release of energy trapped in the stored excited state.

### 4.4 Crystal Structure

#### 4.4.1 Crystal Structure of SAOE

**Figure 24:**
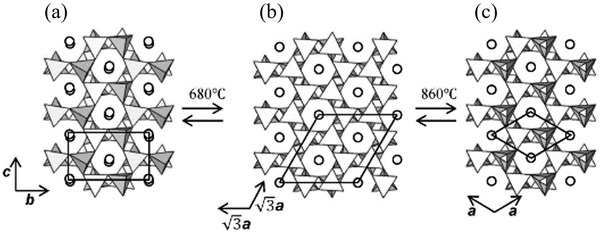
Conversion between three crystal structures at different temperatures: a) monoclinic, b) hexagonal, and c) hexagonal crystal phases. Tetrahedra denote AlO_4_, circles denote Sr, and rectangles and parallelograms denote unit cells. Reproduced with permission.^[^ ^82^ ^]^ Copyright 2012, Elsevier B.V.

SAOE has three crystal structures. The structure of SAOE transforms with temperature, and this transformation is reversible: the monoclinic phase (P21, *α*) at room temperature (**Figure** **24**a), the hexagonal phase (P63(‐A), *β*) at ≈680 °C (Figure 24b), and the hexagonal phase P6322(*β*′) at ≈860 °C (Figure 24c).^[^ ^82^, ^83^ ^]^

When doping, the Eu^2+^ ions (1.12 Å) occupy the sites of some of the Sr^2+^ ions (1.13 Å), and the crystal is not distorted because the radii are similar. If the ionic valence is similar, but its radius and the desired coordination environment are less compatible with the lattice of the matrix compared to Eu^2+^ and Dy^3+^, the luminescent properties, e.g., the afterglow effect, are not as good as this specific ion pair after doping.

#### 4.4.2 Isostructures and Analogs

1) Sr_3_Al_2_O_6_:Eu^3+^/Eu^2+[^ ^8^ ^]^

**Figure 25:**
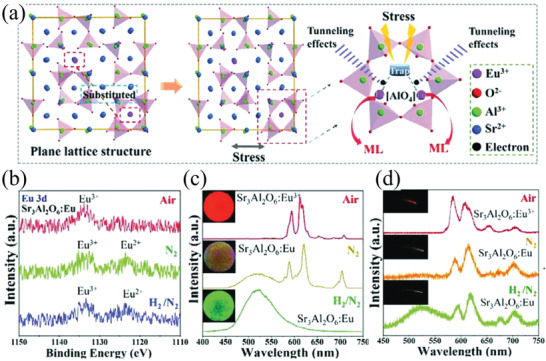
a) Illustration of the structure of the proposed stress‐assisted trap model based on the lattice distortion and tunneling process of ML. b) X‐ray photoelectron spectroscopy (XPS) Eu 3d energy spectra of Sr_3_Al_2_O_6_:8% Eu^3+^ synthesized in different gas environments and c) PL and d) ML spectra after compounding with PDMS. Reproduced with permission.^[^ ^8^ ^]^ Copyright 2018, Wiley‐VCH GmbH & Co. KGaA, Weinheim.

Sr_3_Al_2_O_6_:Eu^3+^/Eu^2+^ was first discovered as an ML material in 1999.^[^ ^84^ ^]^ Wang and co‐workers found that Sr_3_Al_2_O_6_:Eu^3+^/Eu^2+^ calcined in different gas atmospheres would produce different colors during ML/PL (**Figure** **25**a–d).^[^ ^8^ ^]^ When calcined in a N_2_–H_2_ mixture, the Eu^3+^ ions were mostly reduced to Eu^2+^, resulting in green ML. When calcined in air, the Eu^3+^ ions were not reduced, thus retaining the intrinsic red luminescence of Eu^3+^. When calcined in N_2_, some of the Eu^3+^ ions self‐reduced to Eu^2+^, resulting in orange luminescence. The afterglow duration of the three types of luminescence are different; the afterglow duration for green emission is longer, but shorter for red emission.

Sr_3_Al_2_O_6_ (S_3_A_2_O_6_) is easily water decomposable, and even the presence of water vapor in air can make it hydrolyzed. The pseudoperovskite S_3_A_2_O_6_ is usually used as a sacrificial layer to obtain 2D perovskite oxides such as SrTiO_3_ to build van der Waals heterostructures.^[^ ^85^, ^86^, ^87^ ^]^ Usually, water‐inert‐layer coating, such as silicon oxide, yttrium oxide, titanium oxide, aluminum oxide coating and or other stable materials, are used. Oxides and fluorides (e.g., calcium fluoride) can be treated with hydrophobic organics. In short, the water‐splitting problem of S_3_A_2_O_6_ can be addressed.

2) Sr_3_Al_2_O_5_Cl_2_:Ln^3+/2+[^ ^9^, ^88^ ^]^

**Figure 26:**
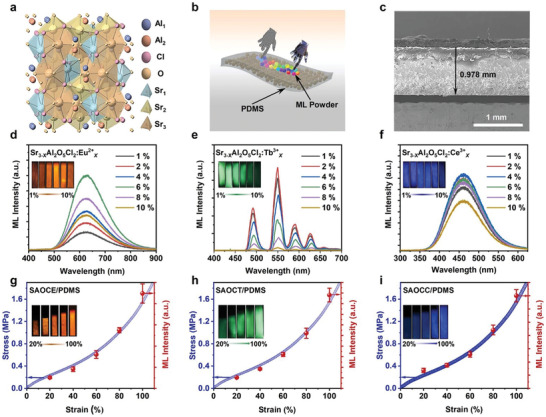
a) Crystal structure of Sr_3_Al_2_O_5_Cl_2_. b) Schematic and c) cross‐sectional SEM image of the ML composite elastomer. d–f) ML spectra of Sr_3_Al_2_O_5_Cl_2_:Ln/PDMS elastomer complexes at different Eu^2+^, Tb^3+^, and Ce^3+^ doping concentrations. g–i) Sr_3_Al_2_O_5_Cl_2_:Ln/PDMS ML intensity versus strain intensity and the amount of stretching at the optimal doping concentrations. The insets show the corresponding ML photographs when subjected to stretching (stretching frequency: 4 Hz). Reproduced with permission.^[^ ^9^ ^]^ Copyright 2022, Elsevier Ltd.

Tang et al. reported that Sr_3_Al_2_O_5_Cl_2_:Eu^2+^ has a peak in the orange broadband PL at 620 nm.^[^ ^29^ ^]^ Wang and co‐workers induced deep traps by codoping 3% Tm^3+^ and 1.5% Eu^2+^, which resulted in a much longer afterglow time (≈220 min).^[^ ^30^ ^]^ Fu and co‐workers found that the ML properties are optimized when codoped with 1.5% Eu^2+^ and 5% Tm^3+^, and a suitable Tm^3+^ concentration (5%) provided deep traps for achieving stable ML performance for a long time even under a load of 10–800 N.^[^ ^88^ ^]^ Nonpiezoelectric (highly symmetric crystals) broad‐spectrum ML was observed for Sr_3_Al_2_O_5_Cl_2_:Ln (Ln = Eu^2+^, Tb^3+^, Ce^3+^) with a spectrum covering blue to orange‐red light (**Figure** **26**a–i), and this ML has a unique temperature‐modulated self‐recovery activity (up to 40% recovery after 30 h at room temperature, 125 °C for 5 min) and good thermal stability.^[^ ^9^ ^]^

In addition, the ML of this material is highly dependent on the substrate material. The ML effect is better when compounded with PDMS and normal when compounded with silica gel. However, there is no ML when compounded with hard epoxy resin. Therefore, it is conjectured that the ML of Sr_3_Al_2_O_5_Cl_2_:Ln requires friction between the ML particles and the polymer, the matrix material to be negatively charged during friction. The Sr_3_Al_2_O_5_Cl_2_:Ln particles do not show ML after they are subjected to the extrusion force of the epoxy resin composite.

3) Sr*_x_* Ca_1‐_ *_x_* Al_2_O_4_^[^ ^89^ ^]^

**Figure 27:**
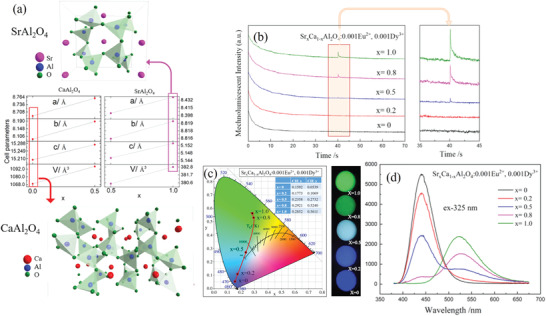
a) Crystal structures and cell parameters of CaAl_2_O_4_ and SrAl_2_O_4_. b) ML intensity excitation spectra of Sr*_x_* Ca_1–_ *_x_* Al_2_O_4_:0.001Eu^2+^, 0.001Dy^3+^ (*x* = 0, 0.2, 0.5, 0.8, 1.0). c) CIE coordinate changes and corresponding photographs. d) Excitation spectra (*λ* = 325 nm, excitation peak of CaAl_2_O_4_). Reproduced with permission.^[^ ^89^ ^]^ Copyright 2022, Wiley‐VCH.

SrAl_2_O_4_ has a similar structure to CaAl_2_O_4_ (**Figure** **27**a), which is also a long afterglow material (emits blue light) but has no ML properties.^[^ ^90^, ^91^ ^]^ Pan and co‐workers found that the PL of SrAl_2_O_4_ can be tuned from green to blue‐green by replacing a portion of Sr atoms with Ca atoms (Figure 27c,d). Although the substitution percentage of Ca can be increased from 0% to 100%, the color during ML remained green, as for SrAl_2_O_4_ (Figure 27b).^[^ ^89^ ^]^

## 5 Synthesis Methods

The most commonly used method for synthesizing SAO is the solid‐phase method. In addition, SAO may be synthesized by laser ablation, the molten salt method and a series of wet chemical methods including the CM, sol–gel method, hydrothermal method, and flame spray pyrolysis (FSP) technique. Most of these methods first synthesize precursor powders and then perform calcination at a high temperature to form target product. As a result of a more complex pretreatment for creating raw materials at the ion level for homogeneous mixing (in contrast to the solid‐phase method with simple mixing of raw materials), the temperature required for calcination is lowered by 100–200 °C or more than the solid‐phase method. Further, the calcined sample particles are more regular, and smaller in size (sub‐micrometers). However, the ML performance is worse for smaller particle sizes owing to the large number of surface defect states. However, ML for nanosized particles is significant for applications such as bioimaging,^[^ ^92^, ^93^ ^]^ printing and textiles. In addition, the presence of a reducing substance or atmosphere is required for all methods to reduce Eu^3+^ and to prevent Eu^2+^ from oxidation. Considering the cost and safety (mainly, the high H_2_ content may cause explosions), the reducing atmosphere is usually provided by activated C or 95% Ar/N_2_ + 5% H_2_. Among these methods, the solid‐phase method is high yielding, simple and producing samples with high luminescent brightness, but energy intensive. The other methods have more complex procedures and lower yields, and most of them require nitrates or raw materials that are hardly available or causing environmental pollution. Therefore, the solid‐phase method is currently mostly used in both laboratory and real‐life production.

### 5.1 Solid‐Phase Method

The solid‐phase method usually produces polycrystalline particles with micrometer size. Although this method is simple, it requires nearly 4 h or more of insulation at 1300 °C or higher. There are issues of energy consumption, the harder lump formation of the product, the complicated grinding process, the irregular shape of the ground powders, and the uniform particle size (a few micrometers to tens of micrometers). Further, agglomerations limit its application in wider fields. Despite the disadvantages of the solid‐phase method, it is still the preferred synthesis method mainly owing to its simplicity of the practice and high yield.

Boric acid is often added as a cofusing agent in the solid‐phase method. Its addition helps lower the reaction temperature, promote the reduction process from Eu^3+^ to Eu^2+^, help Dy^3+^ diffuse uniformly in the lattice, form vacancy defects at suitable depths (which can be excited at room temperature and by small deformations induced with mechanical energy), and enhance the ML brightness and sensitivity. However, the introduction of borate may cause powder agglomerates and hardening, and make it necessary to control the reaction temperature to prevent overburning.

The raw materials routinely used in the solid‐phase method are SrCO_3_, *α*‐Al_2_O_3_, Eu_2_O_3_, and Dy_2_O_3_ (can be replaced with DyCl_3_, chloride also has the ability to aid in melting). If the nitrate decomposition method is used to synthesize the precursor, Eu^2+^ and Dy^3+^ ions are often introduced by Eu(NO_3_)_3_·6H_2_O and Dy(NO_3_)_3_·6H_2_O, respectively.^[^ ^22^ ^]^ H_3_BO_3_ is also often added as a flux to enhance the ML brightness, but the sizes of the sintered particles are larger and difficult to break after its addition.^[^ ^35^, ^42^ ^]^

**Figure 28:**
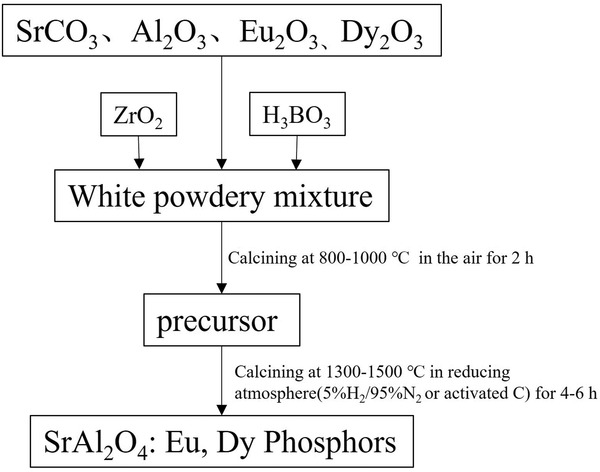
Flowchart for the preparation of SAOED by the solid‐phase method, in which ZrO_2_ and H_3_BO_3_ can be added selectively.

The solid‐phase method can be divided into one or more sintering steps. The two‐step method is more common both in the laboratory and industry. The well mixed raw material is prefired first and then completely react the clinker. In the two‐step synthesis for SAO, the temperature of the first step is controlled at 800–1000 °C for 2 h, and the second step at 1300–1500 °C for 4‐6 h in a reducing atmosphere of 95% Ar/N_2_ + 5% H_2_ or activated C (**Figure** **28**). In addition to direct firing of the powder, the precursor powder can also be pressed into various shapes or coated in different morphologies (e.g., spheres and rods) before firing. However, the temperature is too high, and the fired product is a whole piece of bulk ceramic, not powders.

In industry, ammonia is also commonly used instead of a N_2_–H_2_ gas mixture because liquid ammonia can be directly decomposed to 25% N_2_ and 75% H_2_ with a catalyst when heated to higher than vaporization temperature. This mixture can also provide a suitable reducing atmosphere after further blending. Improvement of the synthesis of SAO can be achieved with the use of organic‐acid‐assisted methods, in which an organic acid promotes better mixing of metal ions with different solubilities that form with SAO. The raw material was all nitrates, and the aqueous solution was mixed well by ultrasound. Malic acid was added at a ratio of 1.5 times the total molar amount of metal cations, and the pH was adjusted to 3 by adding a 28% ammonia solution. The solution was then hot stirred to have water fully evaporated, and a small amount of boric acid was added. The powders were first calcined in air at 1100 °C for 2 h, and then calcined at 1350 °C for 6 h in a 95% Ar + 5% H_2_ reducing atmosphere.^[^ ^24^ ^]^

### 5.2 Laser Vaporization

**Figure 29:**
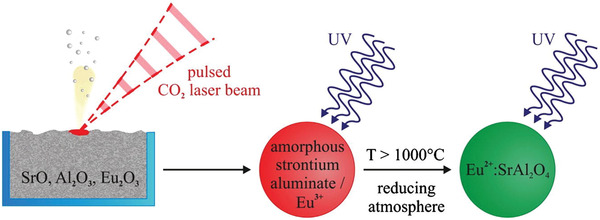
Flowchart for the preparation of SAOED by laser ablation. Reproduced with permission.^[^ ^94^ ^]^ Copyright 2013, Elsevier Ltd.

Laser ablation is another relatively new preparation method (**Figure** **29**). The target SAOED is obtained after calcination. A method of preparation using a CO_2_ laser is as follows. SrO (instead of carbonate), Al_2_O_3_, and Eu_2_O_3_ are mixed in a designed molar ratio and placed in a container, and the laser directly strikes the surface of the mixture to evaporate it after a violent temperature increase to form plasma gas clusters. These clusters are then rapidly cooled to very fine particles in flowing condensing gas and enter a filtering device with the airflow. The nanoscale precursor powders are obtained by extraction, and then calcined in a reducing atmosphere (95% Ar + 5% H_2_) at 1100–1200 °C for 3 h to obtain SAOED.^[^ ^94^ ^]^

### 5.3 Molten Salt Method

**Figure 30:**
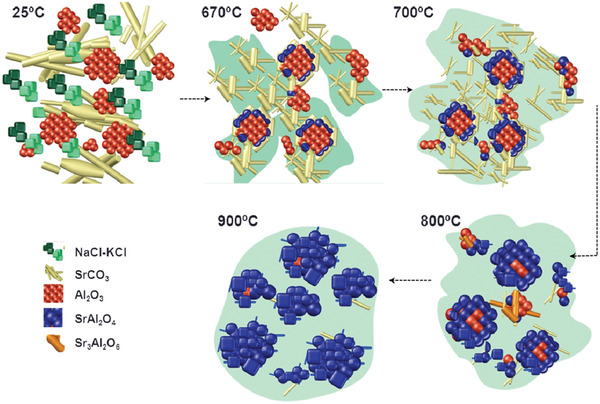
Reaction process of SrAl_2_O_4_. The salt starts to melt at 670 °C. The raw material surrounds the salt, and SrCO_3_ decomposes. Afterward, the temperature continues to increase, and SrAl_2_O_4_ is finally formed. Reproduced with permission.^[^ ^95^ ^]^ Copyright 2015, American Chemical Society.

The molten salt method is similar to the solid‐phase method, but a flux salt is added to the reaction (**Figure** **30**). The molten salt helps the raw material to react more thoroughly, and the final synthesized powder is at the sub‐micrometer with high crystallinity and good luminescence. Salt does not react with the raw materials but will become liquid at high temperatures; then, the raw materials infiltrate it, the ion mobility is greatly increased, the reaction rate accelerates, and the reaction temperature and time are significantly reduced. The raw materials used in the experiment are SrCO_3_, Al_2_O_3_, Eu_2_O_3_, Dy_2_O_3_, NaCl, and KCl. Before the experiment, all raw materials were fully dried in an oven for 1 h. NaCl and KCl at were first mixed at a ratio of 1:1, ground well, then mixed thoroughly at a ratio of 3:1 with SrAl_2_O_4_, and calcined at 900 °C for 1 h in a reducing atmosphere (90% N_2_ + 10% H_2_) to obtain SAOED. After the reaction, the residual remained. Since SAOED reacts with water, the residual salts cannot be removed by water washing either, but this does not greatly affect the luminescent properties of the material.^[^ ^95^ ^]^

The molten salt method is very convenient and low‐cost and may be advantageous in terms of luminescent brightness. For some other oxidation‐prone ML compounds^[^ ^96^, ^97^, ^98^, ^99^ ^]^ such as mass‐prepared sulfides or sulfur oxides, the molten salt method allows some reactions that usually must be carried out in an inert gas to be carried out in air, with reduced costs and effective control over the morphology and size of the crystals.

### 5.4 Combustion Method

Compared with the high temperature and time‐consuming disadvantages of the solid‐phase method, the CM is used to synthesize SAO through the continuous high heat generated by the redox reaction between a metal nitrate and an organic reducing agent (often urea), which can be reacted at 600 °C. The whole process takes a short time, and urea decomposes during the process to produce ammonia as the reducing atmosphere. The particle size is much smaller compared to that prepared by the solid‐phase method, and does not require further ball milling. However, the CM also has the following disadvantages: 1) low yield, 2) flammable and explosive nitrate raw materials, 3) difficult temperature control, and 4) environmental pollution caused by smoke generated. Nitrates can be replaced with metal peraluminate, which releases more heat during the reaction, enhancing diffusion during the reaction.^[^ ^101^ ^]^ Here is an example of a typical process for the preparation of SAO by combustion.^[^ ^23^ ^]^

**Figure 31:**
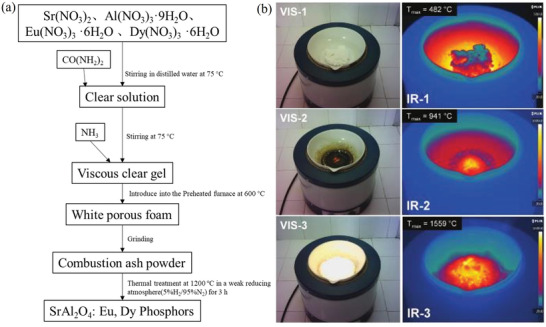
a) Flux diagram for the combustion synthesis method of SAOED.^[^ ^100^ ^]^ b) Still visible (VIS) and infrared (IR) images simultaneously captured during the combustion reaction (1–3). Reproduced with permission.^[^ ^23^ ^]^ Copyright 2016, The Royal Society of Chemistry.

Sr(NO_3_)_2_, Al(NO_3_)_3_·9H_2_O, Eu(NO_3_)_3_·6H_2_O, Dy(NO_3_)_3_·6H_2_O, and CO(NH_2_)_2_ (urea, as a reducing agent) are first mixed, ground for 1 h to make a thick paste, transferred to a crucible, and then calcined at 600 °C. Finally, the sample can be further calcined at a higher temperature in a reducing atmosphere for better crystallinity if necessary. The high temperature makes the mixture to boil, dehydrate, and decompose, which then produces large amounts of gases (carbon oxides, N_2_, and ammonia). The process continues to generate a large amount of heat, and the mixture spontaneously combusts until the temperature threshold is reached and then continues to burn for ≈30 s at temperatures up to 1400–1600 °C (**Figure** **31**b), making the mixture expand, grow larger, and produce white foam and large amounts of ash, which converts the gas‐phase oxides into mixed aluminates. Once the reaction is complete, the crucible can be removed from the furnace and cooled to obtain a fluffy product, which can be gently ground with an agate mortar and pestle to obtain SAO powders (Figure 31a).

### 5.5 Sol–Gel Method

**Figure 32:**
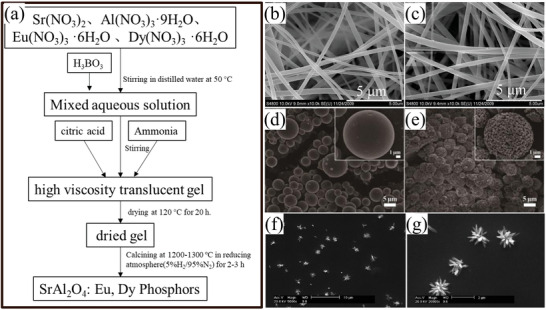
a) Flowchart for the preparation of SAOED by the sol–gel method. SEM photographs of polyvinylpyrrolidone (PVP)/SAOED composite fibers with the addition of b) 0.0075 and c) 0.01125 mol of an inorganic salt. Reproduced with permission.^[^ ^102^ ^]^ Copyright 2010, Elsevier Inc. SEM images of SAOE microspheres: d) dried precursor microspheres and e) caged microspheres after calcination at 1200 °C for 2 h under flowing NH_3_. The insets show the corresponding enlarged SEM images. Reproduced with permission.^[^ ^103^ ^]^ Copyright 2018, The Royal Society of Chemistry. f,g) SEM images of flower‐like SAOED powders at different scales. Reproduced with permission.^[^ ^104^ ^]^ Copyright 2011, The Chinese Society of Rare Earths.

The sol–gel method is also a common method for the synthesis of SAO. It is undeniable that SAO synthesized by sol–gel method has strong ML. Compared with the solid‐phase method, which requires a high temperature and produces particles with nonuniform shapes and large sizes (tens to hundreds of micrometers), sol–gel method can reduce the reaction temperature for obtaining pure‐phase SAO from 1400 to 1200 °C owing to the direct formation of SAO nuclei at the molecular level, followed by calcination with1% boric acid added as cofusing agent. The particles formed are regular in shapes, easy to grind and uniform sub‐micrometer or even nanometer in size. By adding different additives to this method (**Figure** **32**a), fibrous (Figure 32b,c),^[^ ^102^ ^]^ cage‐like (Figure 32d,e),^[^ ^103^ ^]^ and flower‐like (Figure 32f,g)^[^ ^104^ ^]^ SAO particles can be produced.

As the calcination temperature is lower, the crystallinity of the formed powder particles decreases, resulting in smaller particle sizes, more surface defects and thus decreased the luminous intensity and blue‐shifted excitation peak. A typical procedure for this method shows as follows. First, Sr(NO_3_)_2_, Al(NO_3_)_3_·9H_2_O, Eu(NO_3_)_3_·6H_2_O, Dy(NO_3_)_3_·6H_2_O, and a small amount of H_3_BO_3_ are dissolved in DI water at 50 °C, and an aqueous citric acid solution is slowly added and stirred. The pH of the solution is maintained between 2.5 and 3 by adding ammonia. After stirring until homogeneous, the solution is heated to 80 °C and kept stirring until water completely evaporates, and turns into a highly viscous translucent gel. This gel is fully dried at 120 °C for 20 h and calcined at 1200–1300 °C for 2–3 h in a reducing atmosphere (activated C or 95% Ar +5% H_2_).^[^ ^105^, ^106^ ^]^

### 5.6 Hydrothermal Method

**Figure 33:**
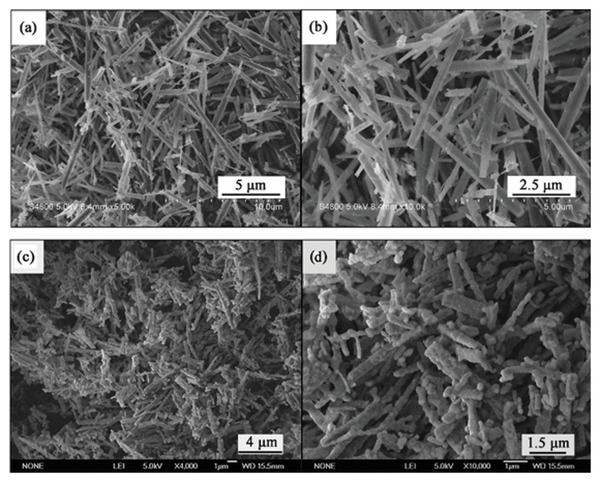
a,b) Field‐emission SEM (FESEM) images of the precursors obtained by hydrothermal treatment at 100 °C for 12 h. c,d) FESEM images of the calcined samples. Reproduced with permission.^[^ ^107^ ^]^ Copyright 2009, American Chemical Society.

The hydrothermal method is similar to the sol–gel method, but this method allows the formation of pine‐needle‐shaped long SAO particles because a typical shape guide, cetyl trimethyl ammonium bromide (CTAB) (**Figure** **33**), is used. The specific preparation method is as follows. An appropriate amount of Sr(NO_3_)_2_, Al(NO_3_)_3_·9H_2_O, Eu(NO_3_)_3_·6H_2_O, Dy(NO_3_)_3_·6H_2_O, CO(NH_2_)_2_, and CTAB are dissolved in DI water and stirred for 30 min. Then, the white suspension is kept in a stainless‐steel autoclave at 100 °C for 12 h and filtered to obtain a white fluffy product. Pine‐needle‐like SAOED is obtained by washing it with DI water and anhydrous ethanol and then drying in vacuum to form the precursor powder (Figure 33), which is then calcined in a reducing atmosphere (95% Ar + 5% H_2_) at 1300 °C for 5 h. The SEM results show that the product is needle‐shaped with diameters of hundreds of nanometers and lengths of tens of micrometers (Figure 33).

### 5.7 Flame Spray Pyrolysis Technique

**Figure 34:**
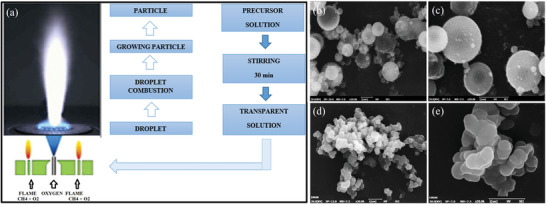
a) Flowchart of FSP. SEM images of b,c) the precursors and d,e) the product obtained by FSP after calcination. Reproduced with permission.^[^ ^108^ ^]^ Copyright 2018, Elsevier B.V.

The FSP technique is a promising method for phosphors synthesis. In this method, precursor solution droplets containing raw materials are instantaneously evaporated with a high‐temperature flame, releasing the precursors. Then, the desired substances are obtained in high‐temperature oxidizing atmosphere. Serdar et al. synthesized SAOED for the first time using the FSP method without using a reducing atmosphere (**Figure** **34**a,d,e).

In the process precursor solutions were separately prepared. Sr(NO_3_)_2_, Al(NO_3_)_3_·9H_2_O, and H_3_BO_3_ were dissolved in DI water, while Eu(NO_3_)_3_·6H_2_O and Dy(NO_3_)_3_·6H_2_O were dissolved in ethanol. Each solution was stirred for 30 min (room temperature) for dissolution. Subsequently, the Sr(NO_3_)_2_ solution was slowly added to the Al(NO_3_)_3_ solution using a syringe to avoid air contact and stirred for 30 min, followed by the addition of a mixture of Eu(NO_3_)_3_·6H_2_O and Dy(NO_3_)_3_·6H_2_O. A boric acid solution and glacial acetic acid (GAA) were then added to form a chelate to obtain the precursor solution (Figure 34b,c). H_3_BO_3_ decomposes into B_2_O_3_ at temperatures up to 300 °C, acting as a reducing agent. They concluded that the theoretical transition of the conformational environment between [BO_3_] and [BO_4_] could enable the reduction of Eu^3+^ to Eu^2+^.^[^ ^108^ ^]^

## 6 Applications

**Figure 35:**
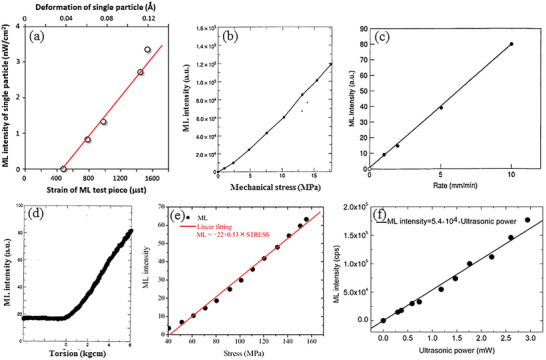
Linear responses of SAOE to different stress stimuli. Reproduced with permission.^[^ ^10^ ^]^ Copyright 2019, Elsevier Ltd.

Chandra proposed that crystals with an exponential distribution of traps would have an exponential relationship between its ML strength the stress, while those with uniformly distributed traps will have its elastic‐mechanoluminescence being linearly related the stress. SAOE has uniformly distributed traps and a good linear ML response to pressure,^[^ ^10^ ^]^ torsional stress, tensile stress, and even ultrasonic forces (force of violent vibration), making it suitable for stress sensors (**Figure** **35**a–f).^[^ ^109^ ^]^ Theoretically, as long as there is deformation, the mechanoluminescence material have potential for ML behavior, the force/pressure threshold value of the prepared ML sensor can be very small. Of course, this depends on the mechanical properties of the ML matrix, the value of Young's modulus, and the sensitivity of the photon detector. Vinogradov and co‐workers reported that a single small SAOE particle (<100 nm) can emit ML light under the action of 10^−17^–10^−16^ N and the pressure of 0.04 Pa, which shows a ultrahigh sensitivity.^[^ ^110^ ^]^ Another determining factor is the nature of the organic matrix used to encapsulate the ML particles. Another report revealed^[^ ^111^ ^]^ that the mechanoluminescence of oxide niobate is a stress‐thresholdless.

### 6.1 Stress Visualization

#### 6.1.1 Stress Monitoring

**Figure 36:**
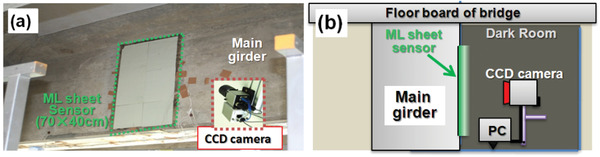
a) Actual diagram and b) schematic diagram of the ML monitoring system. The ML film sensor is attached to the outer wall of the bridge, and a CCD connected to a computer in the darkroom on the right is collecting image data. Reproduced with permission.^[^ ^67^ ^]^ Copyright 2012, SPIE.

ML materials based on SAOE and SAOED can be used for structural health monitoring on the surfaces of and inside buildings,^[^ ^27^, ^112^ ^]^ at pipeline welding points^[^ ^113^, ^114^ ^]^ for high‐pressure vessels,^[^ ^47^ ^]^ etc. The basic idea is to coat the structure to be inspected with an ML composite film; then, images captured with a CCD camera can be investigated in a dark room (**Figure** **36**a,b).^[^ ^115^ ^]^ Capability of this method is limited by the sensitivity of the CCD. Besides, it is difficult to observe very small changes in brightness. Thus, Wang and co‐workers proposed to combine ML materials with distinct stress/strain responsiveness (both color and brightness), achieving different colors of light under different stresses, and the color distinction is easier to be recognized by both human eyes and CCD.^[^ ^116^ ^]^

If repeated and continuous monitoring is required, a UV light source is needed to restore ML properties to the SAO‐based material. As applications broaden, ML materials were also used to monitor the safety of human implants.^[^ ^117^, ^118^ ^]^

**Figure 37:**
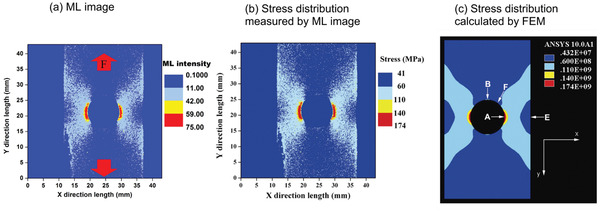
ML technique for visualizing the stress distribution near holes in an Al plate. a) Real‐time ML image of an Al plate. b) Stress distribution map converted from the ML according to a linear relationship. c) Stress distribution calculated by a finite‐element analysis (FEA) method. Reproduced with permission.^[^ ^48^ ^]^ Copyright 2008, The Visualization Society of Japan.

In order to detect the stress on a metal surface, an SAOE film can be pasted onto an Al plate. First, equal weight of SAOE powders and commercial optical epoxy are mixed to prepare an effective ML paste, which is then uniformly applied to the substrate by screen printing at a film thickness of ≈120 µm. The Al plate used for test had dimensions of 225 × 25 × 3 mm^3^ with a 10‐mm‐diameter hole drilled in the center. A 5 kN tensile force was applied every second along the longitudinal direction, and ML was recorded with a high‐speed camera. The location and magnitude of force are determined by the distribution and the intensity of luminescence, respectively. Visualization results show that the stresses on circular sides of the circular hole are more evident, which is different with human intuition (the stresses on the top and bottom sides are higher). This method is more convenient and faster than the optical interference method, showing prospects of SAOE for stress monitoring (**Figure** **37**a–c).

**Figure 38:**
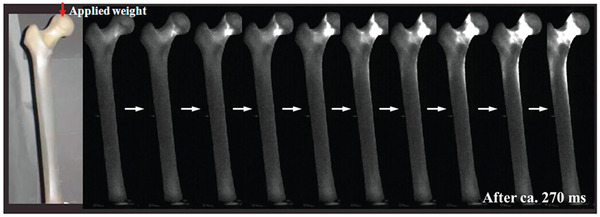
Dynamic ML images of a femur model coated with ML paint under pressure. Reproduced with permission.^[^ ^119^ ^]^ Copyright 2012, Elsevier Ltd.

The surface of an artificial bone can be coated with an SAOE composite, so that stresses on human bone can be visualized using the ML properties of SAOE (**Figure** **38**).^[^ ^117^, ^119^ ^]^ When a load of 100–1800 N is applied to the joint region at a loading rate of 7000 N s^−1^, it was found that the stresses are initially concentrated at the compressed area and then propagate down the femur. This information, which is not visible to naked eyes, helps obtain a better analysis of the abnormal stress concentration prior to fracture.

**Figure 39:**
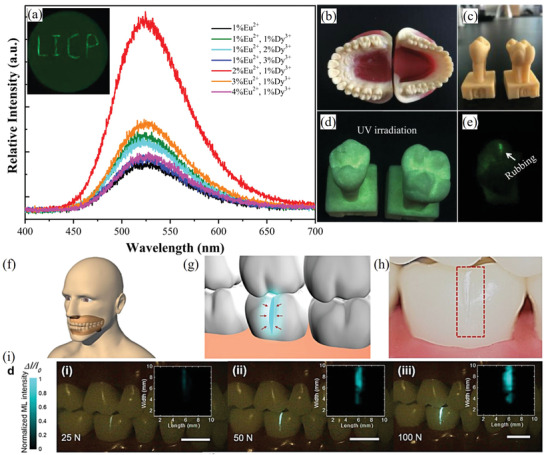
a) ML intensity of SAOED with different Eu^2+^, Dy^3+^ doping concentrations (tested by mixing SAOED and epoxy resin at a weight ratio of 1:7). b,c) Dental molds after compounding this material with DBR. d,e) Luminescence of the composite dental mold under UV and frictional excitation. Reproduced with permission.^[^ ^120^ ^]^ Copyright 2018, Elsevier B.V. f–h) Schematics of the ML particles used to analyze microcracks in teeth. i) Pictures of luminescence at different occlusal forces (dynamic forces) taken by a digital camera. The insets show ML obtained from tooth cracks using the PD array after applying occlusal forces of 25, 50, and 100 N. The scale bar of the white line is 1 cm. The 2D mapping image clearly corresponds to the image captured by the digital camera; thus, it is very convenient to use the PD array instead of the camera for imaging when the mouth is closed. Reproduced with permission.^[^ ^121^ ^]^ Copyright 2022, Springer Nature.

Wang and co‐workers compounded SAOED with a commercial denture base resin (DBR, consists of 99% polymethylmethacrylate powder and 1% benzoyl peroxide) to verify its feasibility to analyze occlusal mechanics of dentures. SAOED has a strong stress‐free afterglow, which may interfere with the analysis of occlusal mechanics using ML. In order to eliminate the effect of the stress‐free afterglow, the concentration of Dy^3+^ is adjusted to be 1%, so that the SAOED had basically no afterglow 20 s after removal of the irradiation source, while its ML intensity was greatly enhanced. The afterglow characteristics of SAOED did not change significantly when the concentration of Eu^2+^ varied from 1% to 4%, and the maximum ML intensity was obtained with a Eu^2+^ doping concentration of 2% (**Figure** **39**a). Thus, SAOED doped with 1% Dy^3+^ and 2% Eu^2+^ were used (Figure 39b–e).^[^ ^120^ ^]^ Subsequent studies found that zirconia ML teeth might be more suitable considering durability and biocompatibility.^[^ ^121^ ^]^ Kim et al. used a self‐healing photodetector array (PD array) to detect tooth microcracks. The ML particles are embedded in the cracks of the tooth model. When chewing, the ML particles are forced to emit light (Figure 39f–h), so that the PD array attached to the tooth generates a photocurrent. The depth, width, length, and position of the microcrack can be obtained accurately by analyzing the photocurrent (Figure 39i). The device maintains a good performance after 50 bites being placed in artificial saliva for 48 h.

**Figure 40:**
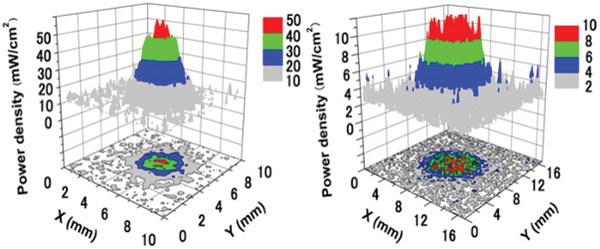
Visualization of the distributions of the output powers of two ultrasonic transducers at 20 MHz (left) and 6 MHz (right).Reproduced with permission.^[^ ^122^ ^]^ Copyright 2011, IOP Publishing Ltd.

It is noted that ultrasound generates vibrations when it propagates through materials and vibrations produce forces. Hence, ML materials can also be used for the visualization of the ultrasonic power (**Figure** **40**), which is useful for medical diagnosis. Xu's group successfully verified the capability of SrAl_2_O_4_:Eu, Ho‐based ML sensors for ultrasonic power measurement and demonstration of output power distribution at frequencies of 6 and 20 MHz.^[^ ^122^ ^]^

#### 6.1.2 Crack Detection

Mechanoluminescent sensors based on SAOE or SAOED can monitor the stress distribution on a solid surface in real time, detect sudden damage (e.g., cracks on the surfaces of pipes, tanks, and bridges), display the stress distribution near a crack tip and calculate crack‐related parameters such as the SIF (stress intensity factor, a parameter to control the stress field near the crack tip). ML materials can be compounded with typical graphite/epoxy resin solid lubricant material, which emits light when the lubricant coating is about to be exhausted, realizing intelligent wear out early‐warning rather than replacing the lubricating coating at a specified service time that causes a great sacrifice on utilization.^[^ ^123^ ^]^ In conclusion, ML‐based intelligent detection can reduce costs and simplify the measurement compared to those of conventional detection methods such as moiré stripes, interferometry, and holography.^[^ ^50^ ^]^

**Figure 41:**
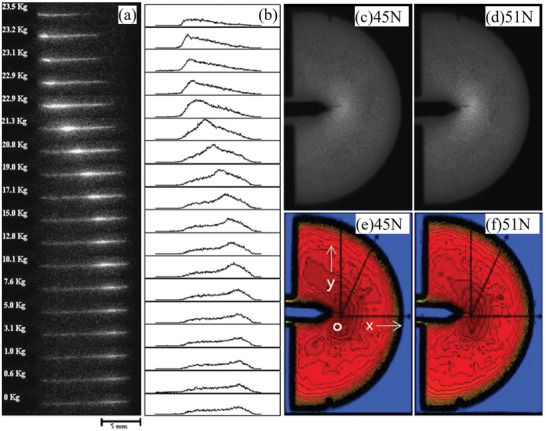
a) ML diagram of the crack extension process. b) Diagram of the stress distribution transformed from (a). Reproduced with permission.^[^ ^124^ ^]^ Copyright 2005, Elsevier Ltd. c–f) ML diagrams of a test disk coated with an ML material under different stresses and the isostress contours calculated from them. Reproduced with permission.^[^ ^50^ ^]^ Copyright 2013, Elsevier Ltd.

Kim and Xu et al. have studied ML visualization of cracks on the surfaces of bridges and tanks. The basic practice is to introduce an artificial crack after coating the specimen surface with an SAO film, mechanically load the crack, record the resulting ML, and then analyze its evolution. Kim et al. proposed a method to measure the crack length during rapid expansion in real time based on SAOED (**Figure** **41**a,b). They mixed epoxy resin and SAOED powder with a 20:3 volume ratio, which is then uniformly coated on an alumina ceramic. The specimen was exposed to UV light (365 nm) for 10 min, and then left in the dark for 5 min to eliminate the afterglow. Subsequently, they introduced a crack opening in the alumina ceramic with a blade, applied a controlled external force to expand the crack by a loading table, and recorded ML images with a high‐speed camera, from which the entire crack path could be clearly observed.^[^ ^124^ ^]^ They also proposed a novel optical method for measuring the type I SIF with an SAOE‐based ML sensor (Figure 41c,d). The experiment was conducted in a dark box. They applied pressure to the half‐disk firmware coated with an ML film. A ML image of the real‐time stress rate near the crack tip of the specimen was recorded with a high‐speed camera and transformed into an isostress contour map, and the SIF was calculated from it.^[^ ^50^ ^]^

**Figure 42:**
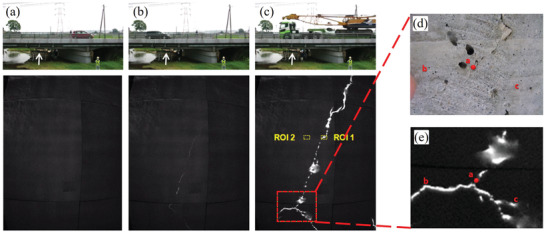
ML‐based active crack monitoring of bridges. ML images of cracks when vehicles pass over the bridge: a) without vehicles, b) with normal vehicles, and c) with heavy vehicles. d) Photograph corresponding to the area in (c). e) ML image corresponding to (d). Reproduced with permission.^[^ ^67^ ^]^ Copyright 2012, SPIE.

Xu et al. produced a system based on SAOE for monitoring bridge construction and demonstrate accuracy, real‐time measurement and reusability (**Figure** **42**a–c). During actual monitoring, they attached several ML films with dimensions of 700 × 400 mm^2^ to a visible crack ≈600 mm long in the bridge and let vehicles pass over this cracked bridge one after another to apply loads. They recorded the ML with a CCD camera, which clearly captured the location and contour of the crack. The displacement of the crack opening was calculated by analyzing ML maps at different moments. Some cracks invisible to naked eye (segments *ab* and *ac* in Figure 42d) can be monitored (Figure 42e), and this ML sensor can last for two years indoors and half a year outdoors.

**Figure 43:**
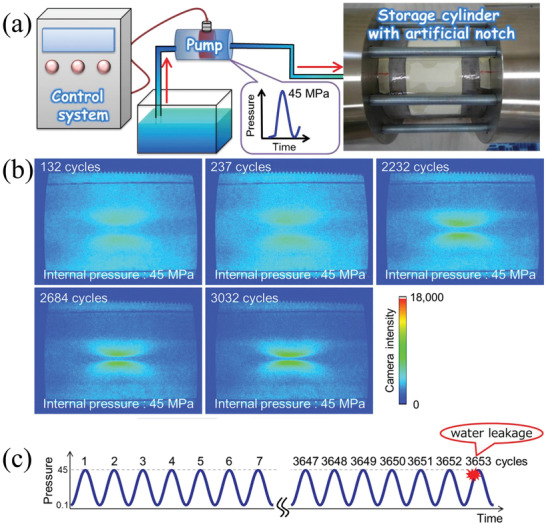
a)Schematic diagram of detecting the surface pressure of the hydrogen storage tanks using the ML films, which will be cyclically applied with pressure from 0 to 45 MPa. b) Images obtained from ML sensor after different numbers of fatigue cycles. c) Rupture of the tank after the 3653rd fatigue cycle and leakage of liquid from the tank. Reproduced with permission.^[^ ^47^ ^]^ Copyright 2015, Hydrogen Energy Publications, LLC. Published by Elsevier.

**Figure 44:**
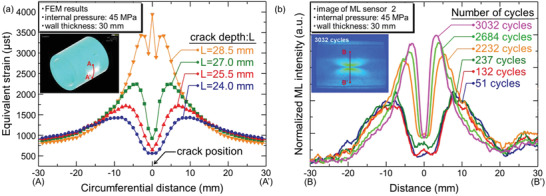
a) Equivalent effect variation curves (line AA′ in the inset) of the storage cylinder model with different crack depths. b) Normalized ML intensity (line BB′ in the inset) for the storage cylinder with artificial notch when the internal pressure is 45 MPa. Reproduced with permission.^[^ ^47^ ^]^ Copyright 2015, Hydrogen Energy Publications, LLC. Published by Elsevier.

ML sensing films can be used for nondestructive crack detection on the internal surface of high‐pressure hydrogen storage tanks (**Figure** **43**a–c). In a study, an artificial U‐shaped notch was introduced to the center of a storage tank as an internal crack. Subsequently, SAOE and epoxy resin mixture was screen printed to form a film. Three sheet ML sensors were fabricated and attached to the external surfaces of a storage cylinder with a commercial adhesive, and ML images were recorded by a CCD camera at a frame rate of 5 fps. Experiments were conducted with cyclic hydraulic pressure from atmospheric pressure to 45 MPa at a frequency of 0.03 Hz, and the changes in the cracks were observed from the ML maps (Figure 43b, **Figure** **44**b).^[^ ^47^ ^]^ The ML diagram also changes during cycling, showing that the changes in ML diagram are correlated with the propagation of the internal crack. Stress analysis of cracks with different depths using a finite‐element method shows that stress concentrated at the tip of cracks with the highest magnitude for the equivalent effect variation (Figure 44a), which is consistent with ML sensor results. The stress concentration gradually decreases as the distance from the crack tip increases, and the equivalent effect variation is symmetrically distributed throughout the crack surface. In addition, the distance between the two ML peaks in the strain map is inverse linearly related to the crack depth, indicating that the ML sensors on the external surfaces can successfully quantify the variations in the internal cracks without loss.

**Figure 45:**
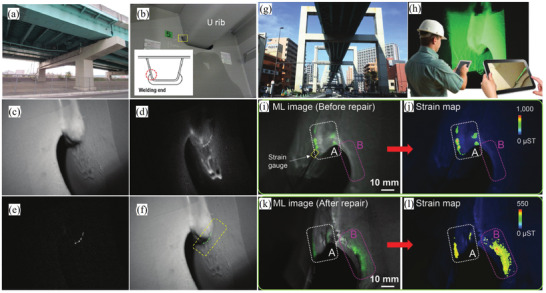
ML method for steel box girders: a) viaduct with box girders, b) test area, c) bright‐field image of the test object, d) ML image after loading, e) image after removing the noise component, and f) synthetic image consisting of (c) and (e). Reproduced with permission.^[^ ^12^ ^]^ Copyright 2022, Fuji Technology Press Ltd. ML inspection of highway bridges: g) monitored highway joints (vulnerable structures), h) simulation of a future inspection scenario.i‐l) ML images and stress concentrations, before i–j) and after k–l) the structure was repaired. The structure was repaired by grinding the welded part using a grinder, which caused the maximum strain to drop from 1000 µST to ≈500 µST. Reproduced with permission.^[^ ^27^ ^]^ Copyright 2018, Wiley‐VCH.

A steel box girder is a common structure used in urban viaducts, and cracks are traditionally detected by the magnetic particle inspection (MT) method. The ML method can detect tiny cracks (<10 mm) that cannot be detected by the MT method and is simpler to operate. In an experiment, a hardened resin containing highly sensitive ML materials was coated to the anticorrosive film around the intersection of the target ribs, and then was heated and dried to produce a 60 µm thick ML sensor (**Figure** **45**a,b). Subsequently, a vehicle load was applied to the target, and images were acquired using a CCD camera and then analyzed (Figure 45c–f).^[^ ^12^ ^]^ After adding Zr ions to obtain a more sensitive ML sensor with and a larger monitoring range, the new sensor was used on a portion of the large infrastructure of the Fukuoka City Expressway (near the entrance to the Golden Bear Interchange) in Japan to perform strain‐accurate imaging, fracture detection and diagnosis to verify its feasibility (Figure 45g–l). Results showed that the method can reduce the cost of crack detection for highway bridges by more than 80% compared to conventional methods such as the MT method.^[^ ^27^ ^]^

### 6.2 ML Skin for Structural Health Monitoring and Human–Machine Communication

SAO can be composited with organic elastomers to form skin‐like patches. Patches can be easily used for long‐distance and large‐area monitoring outdoors and in special structures. However, there are some challenges and uncertainties in the mapping of the ML field to the effective strain field, which limit its significant commercial applications for structural health monitoring systems.

**Figure 46:**
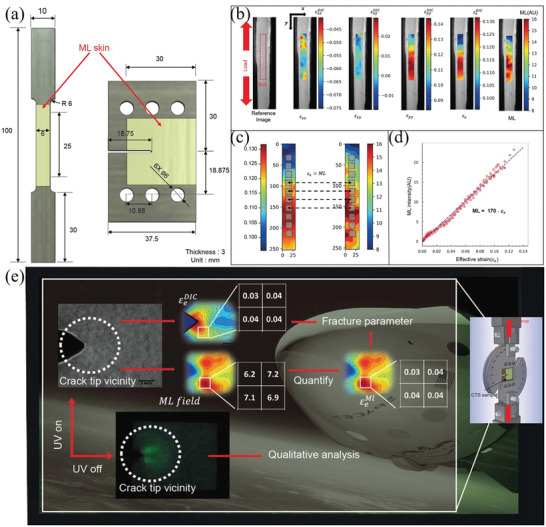
Calibration analysis and demonstration of the strain intensity obtained by the DIC method and ML intensity field. a) Surface‐coated SAOED uniaxial tensile and compact tensile shear (CTS) specimens with a thickness of 80 µm. b) DIC and ML strain analyses of the region of interest (ROI) when the specimen is stretched; from left to right: the image at zero external load; the strain components in the *x* direction, *xy* plane, and *y* direction analyzed by the DIC method when the specimen is loaded; effective strain; and ML intensity field. c) Mapping between the effective strain and the ML intensity field (the last two images in (b)) for different small regions. d) Relationship between the effective strain and the ML intensity obtained by fitting a linear regression model to the scatter. e) Visualization of the ML field and effective strain field of the CTS specimen near the crack tip by DIC and ML. The inset shows the CTS specimen under stress. Reproduced under the terms of the Creative Commons CC‐BY license.^[^ ^125^ ^]^ Copyright 2022, The Authors. Pubished by Wiley‐VCH.

Kim and co‐workers attempted to resolve the quantification problems, and reported that the digital image correlation (DIC) method could be applied to artificial SAOED ML skin to monitor the ML process and quantitatively measure the displacement fields, strain field components and effective strain field (**Figure** **46**a).^[^ ^125^ ^]^ They leveraged the compatibility of ML skin with the DIC algorithm to quantify the ML effects that by using the pixel‐level effective strain and the ML intensity information stored in the same photographic images (Figure 46b,c). Their results show a linear relationship between the effective strain and the ML intensity despite the plastic flow in ML skin (Figure 46d,e). Extension of the DIC method to directly measure the singularity‐dominated effective strain field and determine the structural integrity parameters was crucial in validating measurements using the ML method. Although current work is limited to static cracks, further studies on the evolution of the elastic–plastic field that accompanies advancing cracks during fatigue tests are under investigation.

**Figure 47:**
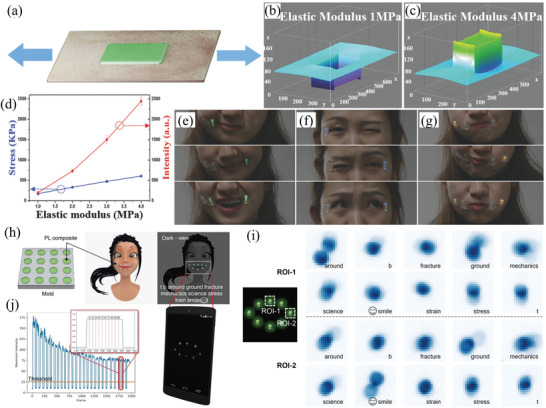
Demonstration of ML‐ or PL‐based skin patches for information exchange. a) Schematic of the ML patch applied to the skin. b,c) Finite Element Analysis (FEAs) for elastic moduli of 1 and 4 MPa. The elastic modulus of human facial skin is 2 MPa, and the elastic modulus of the ML patch needs to be higher than 2 MPa so that the force is mainly concentrated on the ML patch, as shown in (c). d) Relationship between the elastic modulus and the stress from the FEA and the actual ML strength in this experiment. A comprehensive analysis shows that the elastic modulus of the ML patch has a quadratic relationship with the ML strength (red broken line). On the basis of the above analysis, a 200‐µm‐thick ML patch was prepared and attached to the skin in a demonstration, and the movement of muscle for different facial expressions drove the ML patch to emit light at the corners of the e) lips and f) eyes and on g) cheeks. Reproduced with permission.^[^ ^126^ ^]^ Copyright 2018, Wiley‐VCH. h) Schematic of different information conveyed by people using SAOED patches. i) The positional changes result in different information and are highly correlated with the information itself. j) Extraction framework for video information. The raw information in machine learning is frame‐by‐frame pictures taken from a video. A total of 4 × 10 = 40 video clips were obtained from four participants. The information extraction process of each video segment is shown in (j), where the signal continues to decrease owing to the afterglow attenuation of SAOED itself. The inset shows the results of binarizing the 10 frames extracted for each repetition by setting a threshold. Reproduced with permission.^[^ ^127^ ^]^ Copyright 2022, Wiley‐VCH.

SAOED has the characteristics of a long afterglow luminescence and ML. These two characteristics can be independently used to design artificial photonic skin for recording changes in the movement and expression of the human skin or muscles.^[^ ^126^, ^127^ ^]^ This method is expected to be used in human–machine communication and tactile sensing in the future. Li and co‐workers used the sensitive ML properties of ZnS:M^2+^/Cu^2+^@Al_2_O_3_ and SiO_2_ to prepare skin patches in shapes of question marks, exclamation marks, and quotation marks (**Figure** **47**a–d). When the human face makes different expressions, the movement of muscles can drive the skin patch to emit light, presenting a unique effect (Figure 47e–g).^[^ ^126^ ^]^ Kim and co‐workers used the long afterglow property of SAOED in conjunction with Ecoflex and prepared a dot‐shaped patch around the mouth (Figure 47h).^[^ ^127^ ^]^ When a person speaks a specific word (Figure 47i,j), the change in the shape of the human mouth will cause the SAOED patch to move. The position of each patch is located using the afterglow of SAOED, and a convolutional neural network model was trained with information related to the change in position from the patch.

As a result, the specific words expressed by people can be deduced. It has been verified that this method can achieve spoken language prediction correction rates of 98.5% and 98.75% with a visual geometric group network (VGGNET‐5) and residual neural network (ResNet‐34), respectively, and can effectively distinguish similar‐sounding words such as “around” and “ground”. It is noted that the sample size of this experiment is small. There are only seven words, two letters, and a smiling gesture, while larger, broader, and more comprehensive lip language interpretation needs further research. Nevertheless, there is no doubt that this experiment provides a new strategy for lip language interpretion in low‐light environments and realizing human–computer interaction under complex conditions.

### 6.3 Demonstration of Static Electricity

**Figure 48:**
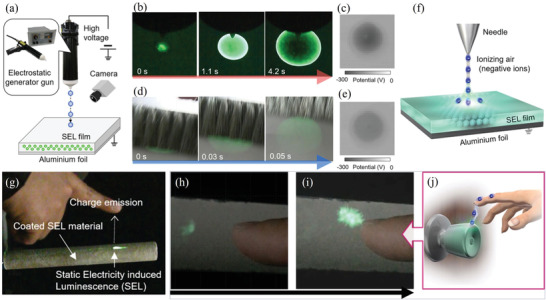
a) Device for measuring the electrostatically induced luminescence of SAOE. b) Observations of light emission that diffuses from the central region of the SAOE film corresponding to the tip after using an electrostatic generator to charge the SAOE film. c) Potential distribution on the upper surface of the SAOE film after charging. d) Discharge of the SAOE film using an antistatic brush. e) Potential distribution on the upper surface of the SAOE film after discharging. f) Movement of ions during the charging process. The electrostatic generator ionizes the air near the needle tip, and negative ions are emitted from the needle electrode and incident on the SAOE film. The negative ions include OH^−^, NO^3−^, and HCO_3_^−^, which collide with the SAOE film to form negative charges and induce SAOE to emit light. g) Discharge of an electrostatic generator coated with an SAOE thin film via a finger. This process can induce SAOE to emit light. h,i) Light emitted when a finger with a voltage of 3–4 kV is brought close to the SAOE‐coated aluminum foil (1–2 cm). j) In some scenarios that are prone to static electricity, this light emission can act as a reminder. Reproduced with permission.^[^ ^128^ ^]^ Copyright 2022, Springer Nature.

Kikunaga and Terasaki reported that electric charge could be visualized using SAOE powders. A small electrostatic sensor emits light when charge is injected(noncontact, **Figure** **48**a), SAOE will form a strong electric field. This electric field is variable and either attenuated or enhanced depending on the distance between these charges (Figure 48g–i), an effect similar to AC EL.^[^ ^81^ ^]^ In fact, this static electricity can automatically form in the environment or be artificially injected, e.g., by the power supply of an electrostatic generator (Figure 48b–f). The distribution of the static electricity of a 3D object could be mapped remotely in real‐time by EL images acquired from a digital camera. This method enables easy visible recognition of noncontact and remote charges by taking advantage of the AC EL properties of SAOE (Figure 48j).^[^ ^128^ ^]^

### 6.4 Advanced Lighting, Imaging, and Anticounterfeiting

**Figure 49:**
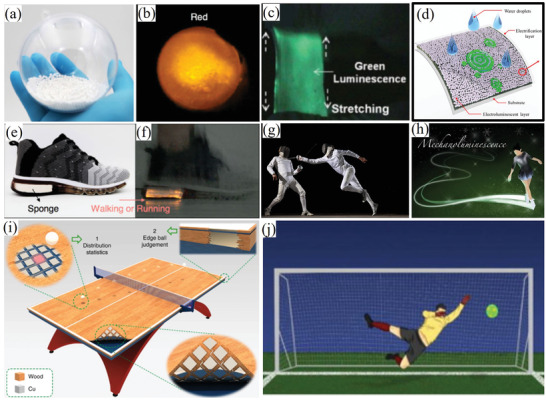
Some application scenarios based on ML and EL. a,b) ML ball driven by ocean energy. Reproduced with permission.^[^ ^33^ ^]^ Copyright 2021, UESTC and John Wiley & Sons Australia, Ltd. c) Stress detection film. Reproduced with permission.^[^ ^139^ ^]^ Copyright 2013, AIP Publishing. d) Novel electroluminescent material based on the triboelectric field at the solid–liquid interface. Light emission occurs when a droplet strikes the surface of the device. Reproduced with permission.^[^ ^140^ ^]^ Copyright 2020, Elsevier Ltd. e,f) Light‐emitting sneakers. Reproduced with permission.^[^ ^33^ ^]^ Copyright 2021, UESTC and John Wiley & Sons Australia, Ltd. g) Artificial visualization of the future use of ML materials to determine the scoring area in fencing. h) Schematic diagram of the use of ML materials for snow and ice sports. i) Smart training table for table tennis based on triboelectric nanogenerator. We can combine the triboelectric nanogenerator with ML materials to display the landing point of the ball.^[^ ^141^ ^]^ j) Display of a stress‐induced glowing soccer ball in a soccer sports scene. Reproduced with permission.^[^ ^141^ ^]^ Copyright 2020, Science China Press.

In addition to classical applications such as stress sensing and visualization mentioned above, SAOE with EL, AL, and ML properties can be employed in other innovative applications (**Figure** **49**). In addition, SAOE's ML has characteristic green light, compounding with ML materials having different colors, such as ZnS:Mn or CaZnOS:Mn (orange‐red light) or other stress‐induced luminescent materials will render promising applications.^[^ ^129^, ^130^, ^131^, ^132^, ^133^, ^134^ ^]^ ML materials can also be used as an emergency lighting energy source (Figure 49a–d). In sports training, it can be used in shoes (Figure 49e,f), art of fencing (Figure 49g), diving,^[^ ^135^ ^]^ skating (Figure 49h), ball sports such as squash, ping pong (Figure 49i), and soccer (Figure 49j) to display points at which balls strike a surface or goal and used in fencing to visualize the point of a sword in order to improve judgment of the fencing area to calculate the score. ML materials compounded on the skateboard or on the ice surface show very beautiful curves during the sliding and improve the visual effect (Figure 49h).

The drum membrane of a drum kit or the outer edge of a drumstick can be coated with these ML particles. When the drum head is struck, it undulates and emits light with the undulation of the head. Through this light emission via ML, the sound and vibration^[^ ^112^ ^]^ can be turned into dynamic light, allowing visual sensing of music by deaf people. It could be implanted into glass for sound field detection and mapping.^[^ ^136^, ^137^ ^]^ At the same time, ultrafine ML particles can be used for biological sono‐optogenetics and temperature imaging.^[^ ^93^, ^138^ ^]^

ML beads with different emission colors can be in situ‐grown on small zirconia balls and then encaged in large plastic ball. At search and rescue sites such as jungles and seas, the shaking produced by humans or sea waves can cause the beads inside to collide and rub against each other to emit light, which indicate, guide, and send distress signals. In terms of anticounterfeiting, convenient, effective, and low‐cost anticounterfeiting coatings based on ML are able to produce luminescent patterns or logos when scratched, and can be used with existing fluorescent and 2D/3D code anticounterfeiting techniques.

**Figure 50:**
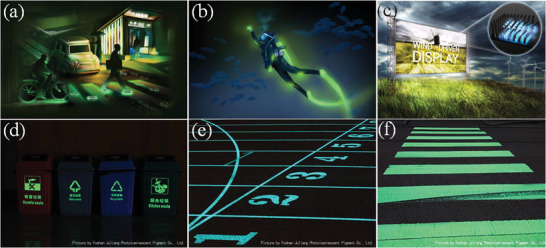
a) Schematic diagram of a smart city with ML roads paved. b) Schematic illustration of a wearable device fabricated using ML smart materials for diving exercise. c) Wind‐driven display panel based on a stress‐induced light‐emitting display unit. c) Reproduced with permission.^[^ ^142^ ^]^ Copyright 2014, The Royal Society of Chemistry. d–f) Fluorescent signs, runways and sidewalks. Reproduced with permission from Foshan Juliang Photoluminescent Pigment Co., Ltd.

At present, SAOED has been widely used in the manufacturing of fluorescent traffic signs,^[^ ^143^ ^]^ including fire escape signs, road signs, and roadside guide markers (**Figure** **50**). Pavement made of fluorescent SAOED can partially convert heat into light, lowering the surface temperature of concrete roads by up to 3–4 °C, while emitting fluorescence at night.^[^ ^144^ ^]^ However, these current applications mostly use the photoluminescence properties of aluminates. If the properties of ML are fully applied into in the field of road lighting, it may bring significant opportunities in the fields of energy saving and traffic safety (Figure 50a), such as visualizing of walking and the trajectory of vehicles, playing the role of vehicle warning, forming a unique urban landscape (Figure 50d–f). In sports science, ML materials can be used in various scenarios (Figure 50b), such as achieving motion‐driven lighting, visualizing the sports intensity (forces), and locating movement trajectory. Advanced displays based on ML materials can also harvest natural mechanical energy such as wind energy (Figure 50c) and liquid flow energy, which as an alternative will help resolve the contradiction between energy shortage, high electricity demand and serious light pollution in our future smart cities.^[^ ^145^ ^]^

**Figure 51:**
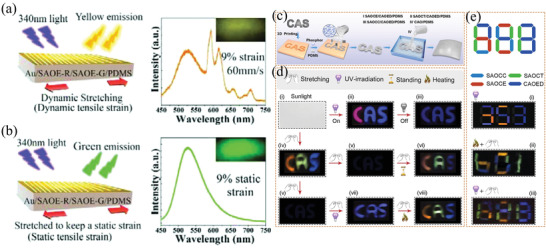
a) Yellowing of the composite film under dynamic tensile strain (dynamic tensile strain: 9%; stretching speed60 mm s^−1^). b) Green afterglow of the laminated film after the stretching stops. Reproduced with permission.^[^ ^8^ ^]^ Copyright 2018, Wiley‐VCH. c) Manufacturing process for multimodal anticounterfeiting devices. d) Optical photographs of multimodal anticounterfeiting patterns under stimuli such as force/light/heat. e) Optical photographs of anticounterfeiting devices capable of displaying different information under different combinations of stimuli. Reproduced with permission.^[^ ^9^ ^]^ Copyright 2022, Elsevier Ltd.

By combining the luminescent properties of different materials, novel and practical anticounterfeiting methods can be developed. For example, a dual‐responsive anticounterfeiting flexible device is prepared using Sr_3_Al_2_O_6_:Eu^3+^(SAOE‐R) and Sr_3_Al_2_O_6_:Eu^2+^(SAOE‐G). A stretchable flexible film coated with a thin layer of Au on the surface to attenuate the green afterglow of sample and ensure that the ML is easier to be observe. The red and green luminescence mixed under dynamic stretching, resulting in yellow light (**Figure** **51**a). When stretching is stopped, the film emits green afterglow because the red portion is basically free of afterglow (Figure 51b). These properties can be employed to distinguish dynamic and static stresses. Besides, a multimodal anticounterfeiting device was fabricated by a simple permutation method, in which three materials were mixed with the conventional blue phosphors CaAl_2_O_4_:Eu/Dy and then compounded with PDMS to form the letters “CAS,” so that each letter has different PL, ML, and afterglow (**Table** **2**). After excitation with UV light with a wavelength of 365 nm (the Sr_3_Al_2_O_5_Cl_2_:Tb^3+^ excitation peak is at 291 nm, and does not match the 365 nm light, so it shows weak PL), different information can be displayed in different states such as static, dynamic stretching and stretching termination (Figure 51c,d).

**Table 2:** ML and PL of four materials that make up the letters “CAS”

Material	ML Color	PL Wavelength
Sr_3_Al_2_O_5_Cl_2_:Eu^2+^ (SAOCE)	Orange‐red	*λ*_ex_ = 358 nm, *λ*_em_ = 626 nm (orange‐red)
Sr_3_Al_2_O_5_Cl_2_:Tb^3+^ (SAOCT)	Green	*λ*_ex_ = 291 nm, *λ*_em_ = 547 nm (green)
Sr_3_Al_2_O_5_Cl_2_:Ce^3+^ (SAOCC)	Blue‐violet	*λ*_ex_ = 338 nm, *λ*_em_ = 449 nm (blue‐violet)
CaAl_2_O_4_:Eu/Dy (CAOED)	–	*λ*_ex_ = 325 nm, *λ*_em_ = 442 nm (blue‐violet)

### 6.5 Wearable Fabrics

**Figure 52:**
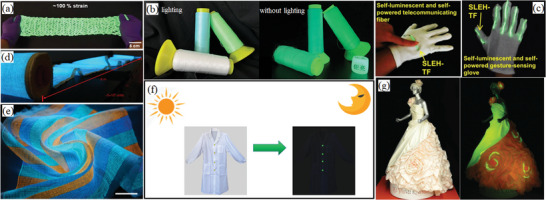
a) SAOE composite fabric combined with nanogenerator. Reproduced with permission.^[^ ^147^ ^]^ Copyright 2022, Elsevier Ltd. b) SAOED could be incorperated into yarn^[^ ^148^ ^]^, the commercial shown with lights on and off. Reproduced with permission from Foshan Juliang Photoluminescent Pigment c) Wearable fibers combining a nanofrictional luminescence machine and the EL properties of SAOE for generating Morse code and sensing human gestures. Reproduced with permission.^[^ ^147^ ^]^ Copyright 2022, Elsevier Ltd. d,e) Fabrics based on the EL properties of ZnS. Reproduced with permission.^[^ ^149^ ^]^ Copyright 2021, Springer Nature. f) Fluorescent buttons based on SrAl_2_O_4_:Eu^2+^/Dy^3+^. Reproduced with permission.^[^ ^150^ ^]^ Copyright 2021, Elsevier Ltd and Techna Group S.r.l. g) Flexible fluorescent clothes prepared from luminescent silk. Reproduced with permission.^[^ ^146^ ^]^ Copyright 2013, Wiley‐VCH GmbH.

However, the long afterglow properties of fluorescent materials, such as SrAl_2_O_4_:Eu^2+^/Dy^3+^ and Sr_2_MgSi_2_O_7_:Eu^2+^/Dy^3+^, combined with the electroluminescent ZnS supplied by battery, electro‐optical conversion or force‐electro‐optical conversion, offers a strategy for future application of ML in wearable devices. Specifically, fluorescent powders are deposited onto silk threads, buttons or other clothing materials to achieve fluorescence on clothing (**Figure** **52**).

A nanogenerator, generating power from friction between clothing and human body during movement, combined with autoluminescent SAOE to display information on clothing (Figure 52a,c). ZnS particles are directly compounded with clothing fibers after a waterproof treatment, and information is displayed on the clothing through an on–off circuit (Figure 52d,e). A Japanese group prepared transgenic silks in green, red, and orange fluorescent colors using transgenic silkworms, and clothes spun from the transgenic silks had a gorgeous shimmering effect (Figure 52g).^[^ ^146^ ^]^ It is possible to realize tactile feedback and the display of information on clothing, seats, and even artificial electronic skin if ML materials are compounded.

It should be noted that some stretchable large‐area ML devices are fabricated from commercial ZnS:Mn/Cu phosphors. The main difference between the ZnS and SAO is that the former does not require pre‐irradiation; while the latter need supplemental light source as an aid to sustain mechanoluminescence. It can be used along with near‐ultraviolet or even blue LED to realize the enhancement of mechanoluminescence (Figure 4b). In addition, by changing the structures/orientations of the SAO host, applying the stress in the form of friction, which can make it possible to improve the repeatability of mechanoluminescence. It is worth noting that in addition to strontium aluminate, other mechanoluminescent materials are also emerging, such as organic materials,^[^ ^151^, ^152^ ^]^ biohybrid systems,^[^ ^153^ ^]^ and other hosts such as inorganic materials, such as low‐cost phosphates.^[^ ^116^, ^154^, ^155^, ^156^ ^]^ It is believed that more and more ML materials will be developed in the future, which will provide a library for understanding and analyzing the mechanism of mechanoluminescence and realizing practical applications.

## 7 Summary and Outlook

SAO is a classical mechanoluminescent material, and great progress on the mechanism, synthesis methods, and characterization of SAO has been made since the discovery of its ML properties. The enthusiasm for developing force sensors and other commercial applications using its ML properties keeps blooming, but there are obstacles hindering its broad applications at this time, and possible solutions to those obstacles are discussed as follows.

1) Improving understanding on the ML mechanisms of SAO. At present, the piezoelectric model is generally accepted, in which Eu acts an activator, but the role of Dy remains unclear. Some researchers claim that Dy increases the number of traps at moderate depths, while others claim Dy facilitate the increase amount of traps near Eu. Improvement in brightness by simple ion doping has reached a bottleneck partially because of the unclear mechanism. SAO is a light‐storage‐light‐release‐type material. A force excites a piezoelectric field, the electrons in traps are released and undergo stimulated radiation before producing ML. At room temperature, these electrons can also be spontaneously excited to produce an afterglow. In this mechanism, UV light is needed to charge SAO, but UV light inevitably induce an afterglow. One solution is to avoid the use of UV light and introduce new defects(by doping with specific ions) instead，allowing SAO to absorb and store a wider band of electromagnetic waves in the new defects, which is similar to the original SAO sensors powered by batteries. Better energy supply without leakage is highly desired for stronger and stabler ML. Therefore, in‐depth understanding of the ML mechanisms of SAO is crucial to further development and application of high‐performance SAO.
2) Improving the ML brightness limit, sensitivity and repeatability of SAO and moderating afterglow. Breakthroughs in these properties can be achieved by ion doping, process optimization or heterojunctions. There have been considerable attempts at ion doping, but for ML at yellow‐green wavelengths, no other ions have been found to be able to significantly improve the brightness except Eu^2+^ and Dy^3+^, and the Dy^3+^ will lead to the synchronized enhancement of ML and afterglow, which will cause interference in the field of sensing but be beneficial for electricity‐saving light sources. ML at near‐UV and NIR wavelengths has been achieved by doping with Cr^3+^ and Nd^3+^ plasma, but the light intensity is relative weak. There are still many challenges to overcome to obtain ML materials with a brightness comparable to that of LEDs, but the highest intensity of ML materials developed so far has reached ≈200 cd m^−2^, which is enough for emergency lighting.
3) Improving the structural stability of SAO. Existing solution to resolve the structural instability of SAO is to protect SAO particles by coating, but it does not guarantee effectiveness or effective transmission of force when subjected to a stress. Therefore, more effective and durable protection schemes besides of simple coating or compositing with PDMS, epoxy resins, and other materials need to be developed to meet the versatile requirements of SAO in various application scenarios and there should also be a set of feasible criteria to test its service life under specific usage scenarios, such as quantification issues, life time, light decay, etc.
4) Preparation of nanoscale, morphologically controlled and uniformly‐size SAO particles. Current methods in preparing SAO of nanoscale and various shapes have low‐yield, and produces smaller particles leading to decrease in luminous intensity. SAO ML particles prepared by the conventional simple solid‐phase method are large, different in size and irregular in shapes, but they do work well in terms of the luminous intensity. We need to find a strategy to reduce the size of the SAO particles to smaller scale without significantly sacrificing their luminescence, and explore more convenient methods to prepare monodispersed SAO in large quantities, in order to satisfy specific size applications, such as 3D printing structures, wearable fiber fabrics and advanced soft light‐emitting devices.
5) Techniques for characterization of the uniformity of ML brightness and mechanical‐to‐optical energy conversion. Current techniques of sample preparation and ML intensity characterization vary among teams. The preparation process of test samples, selection and ratio of compositing materials, and sample thickness will cause subjective differences in brightness. Different test methods, such as impact, circle, and tensile test, vary in the results obtained by different groups. A set of feasible and accurate measurement methods is essential. For different mechanoluminescence forms, such as deformation‐ML, tribo‐ML, etc., different measurement methods, including single particle measurement, in situ microscopy, integrated electron, and optical microscopy, should be adopted. In addition, taking into account the requirements of different usage scenarios, the test standards should include threshold pressure and saturation stress loading tests. Standard characterization techniques and evaluation terms need to be established for better comparison and assessment of materials reported.
6) ML materials have great development potential in energy, sensing, information, and other fields. For the stress/strain monitoring of bridges, steel structures, and precision components, ML coatings should be designed to achieve nondestructive and high‐precision detection; for outdoor emergency, different mechanical energy excitation light‐emitting devices can be designed to realize the collection and utilization of wind energy, water energy and sound energy; for emergency situations such as fire, lightweight SAO aerogels can be made into waterproof and fireproof carpets. Therefore, products based on ML materials are urgently needed to meet the requirements from actual production and life. In large‐scale applications, it is also necessary to formulate standards for detection methods and signal extraction. The signal end requires further development of painting, coating and spraying techniques to prepare uniform ML film to achieve the unification of optical signals; the transmission end can integrate the signal end with optical fiber and ultrasensitive photoelectric detection equipment such as CCD and CMOS, and the receiving end needs to ensure that ML signals are correctly quantitatively mapped and analyzed to achieve the precise and accurate engineering measurements.

SAO is not a particularly new light‐emitting material, but the study and use of its ML light‐emitting properties are still at the preliminary stage. Even though we have been studying SAO for decades (from long afterglow materials onward), it is still difficult to put it into practical use considering the cost of preparation, the luminescent efficiency, stability and so on. However, among all the stress‐induced light‐emitting materials, SAO still has many incomparable advantages: 1) Convenient to be charged. It can be charged by sunlight, LEDs and all types of artificial light sources, which are much more convenient than ML materials need to be charged with *γ* rays. 2) Maturely‐developed. As a mature commercial phosphor, SAO has a broad industrial base, so it can achieve rapid industrialization by upgrading existing production lines after finding feasible ways to enhance the performance of ML. Importantly, SAO is safe and existing as a natural mineral, containing no heavy metals, so there is no pollution problem when it is released nature.

On the basis of all these considerations, we believe that SAO is one of the most promising smart and marketable stress‐induced light‐emitting materials. We hope it can provide unique value in various fields, such as auxiliary lighting, new energy, stress monitoring, motion analysis and anticounterfeiting, to help build smarter, more beautiful and more environmental friendly cities.

## Conflict of Interest

The authors declare no conflict of interest.
